# Cluster analysis of conformity and social desirability: Association with use and problematic use of licit substances

**DOI:** 10.1016/j.abrep.2026.100710

**Published:** 2026-05-20

**Authors:** Rana Hatem, Sandrella Bou Malhab, Chadia Haddad, Hala Sacre, Aline Hajj, Fouad Sakr, Roy Eid, Georgia Chkayban, Clara Gerges, David Karam, Marguerite Ghostin, Dalal Youssef, Pascale Salameh

**Affiliations:** a Institut National de Santé Publique, d'Épidémiologie Clinique et de Toxicologie-Liban (INSPECT-LB), Beirut, Lebanon; b Gilbert and Marie-Rose Chagoury School of Medicine, Lebanese American University, Byblos, Lebanon; c Research Department, Psychiatric Hospital of the Cross, Jall Eddib, Lebanon; d Faculty of Public Health, Lebanese University, Fanar, Lebanon; e Inserm U1094, IRD UMR270, Univ. Limoges, CHU Limoges, EpiMaCT - Epidemiology of Chronic Diseases in Tropical Zone, Institute of Epidemiology and Global Health – Michel Dumas, OmegaHealth, Limoges, France; f Drug Information Center, Order of Pharmacists of Lebanon, Beirut, Lebanon; g Faculté de Pharmacie, Université Laval, Québec City, Québec, Canada; h Oncology Division, CHU de Québec-Université Laval Research Center, Québec City, Québec, Canada; i School of Pharmacy, Lebanese International University, Beirut, Lebanon; j Faculty of Medical Sciences, Lebanese University, Hadat, Lebanon; k Department of Psychology, Bordeaux University, Bordeaux, France; l Faculty of Medicine and Medical Sciences, University of Balamand, Beirut, Lebanon; m Faculty of Medicine, Paris-Saclay University, le Kremlin-Bicêtre, France; n Lebanese Higher Institute of Technical & Professional Education (IPNET), Beirut, Lebanon; o Faculty of Pharmacy, Lebanese University, Hadat, Lebanon; p Department of Primary Care and Population Health, University of Nicosia Medical School, 2417 Nicosia, Cyprus

**Keywords:** Problematic substance use, Social desirability, Profiling, Cigarette, Waterpipe, Alcohol

## Abstract

**Background:**

Problematic Use of Licit Substances is an increasing public health concern in Lebanon, where political and economic instability, alongside cultural and religious diversity, may intensify risk behaviors, while social desirability and conformity can influence their reporting. This study aims to assess the profiles of participants related to social desirability and conformity pressures and their association with licit substance use and problematic use.

**Methods:**

A cross-sectional online survey was conducted among 460 adults in Lebanon (February–March 2025). Data on cigarette, waterpipe, and alcohol use were collected alongside validated measures of social desirability and conformity. A two-step cluster analysis was first conducted to determine the optimal number of clusters, followed by K-means clustering to classify participants based on their social desirability and conformity scores, with subsequent regression analyses examining sociodemographic and cluster membership correlation with substance use behaviors

**Results:**

Three clusters emerged: Norm Internalizing Perfectionists (*N* = 127 (29.2%)); Detached Autonomists (*N* = 143 (32.9%)); and Strategic Conformists (*N* = 165 (37.9%)). The risk of problematic substance use was higher in the detached/autonomous phenotype and increased with age and among male gender participants, while religious prohibition appeared strongly protective. Differential results were found for waterpipe and alcohol, in comparison with problematic cigarette use

**Conclusion:**

Considering social desirability and conformity allows for more precise identification of problematic substance use clusters, guiding targeted prevention and intervention. This complex interplay of personality traits, cultural values, age, gender, and education highlights the need for culturally sensitive, gender-specific, and cluster-tailored public health and psychosocial interventions rather than universal approaches.

## Highlights

• Cluster analysis can reveal profiles linking social and psychological factors.
• Detached Autonomists had highest cigarette and waterpipe dependence.
• Perfectionists and Conformists had lower waterpipe dependence.
• Religious alcohol prohibition was associated to lower alcohol use.
• Three profiles show personality and norms shape risk behaviors.

## 1 Introduction

Problematic use of licit substances affects over 296 million people worldwide, posing a major global public health concern (UNODC, 2023). This complex issue spans biological, psychological, and sociocultural domains (World Health Organization, 2019). The Eastern Mediterranean Region (EMR) faces particularly high rates of waterpipe smoking, especially among youth, yet research on substance use patterns in the Middle East and North Africa (MENA) region remains limited (Darijani et al., 2025; Jawad and waterpipe tobacco knowledge hub, 2018; Nakkash et al., 2022). In Lebanon, political and economic instability has exacerbated problematic substance use, with treatment centers reporting a 169% rise in substance use disorder cases between 2019 and 2022 (Gibon, 2023). Current data show high prevalence rates: cigarette smoking affects 48.6% of males and 21.5% of females, waterpipe use 32.7% and 46.2%, respectively, and alcohol use disorder impacts about 11% of adults (Nakkash et al., 2022; Yazbek et al., 2014).

Traditional analyses that assess substances separately overlook the complex clustering of behaviors. Cluster analysis offers a comprehensive approach to identify user profiles reflecting social, demographic, and psychological factors, supporting more targeted prevention strategies (Charron et al., 2023). Research conducted across diverse cultural and geographical settings has highlighted the critical role of both social desirability and conformity in shaping substance use behaviors and their measurement. Social desirability bias, defined as the tendency to report behaviors in alignment with perceived social expectations, has been consistently associated with the underreporting of stigmatized behaviors such as alcohol consumption, smoking, and illicit drug use, particularly in settings where these behaviors are socially or culturally discouraged (Latkin et al., 2017, Vergés, 2022; Singh et al., 2022). This bias appears especially pronounced in conservative or collectivist societies, where adherence to social norms is highly valued (Alhashimi, Khabour, Alzoubi, & Al-shatnawi, 2018; Caputo, 2017). Conformity, in parallel, has been widely identified as a key psychosocial factor influencing substance use initiation and maintenance, particularly among adolescents and young adults (Schulenberg, Maslowsky, & Jager, 2018). Individuals with higher conformity tendencies are more likely to adopt behaviors aligned with group norms, including smoking or alcohol use in peer environments where such behaviors are socially accepted (Bobo & Husten, 2000; Esteban-Gonzalo, Sik Ying Ho, Aparicio-García, & Esteban-Gonzalo, 2020).

In Lebanon's collectivist society, strong family and community ties heighten the influence of social desirability and conformity on substance use (Caputo, 2017; Ministry of Public Health, Ministry of Education and Higher Education, Ministry of Interior and Municipalities, Ministry of Justice, and Ministry of Social Affairs, 2016). Cultural and religious norms shape acceptance levels, with waterpipe smoking often viewed as more acceptable than cigarettes (Salameh et al., 2014). Yet, Lebanese research rarely applies analytical methods that capture the interplay of structural, cultural, and psychosocial factors. Using cluster analysis, as shown elsewhere (Charron et al., 2023; Panlilio et al., 2020), could deepen understanding of these dynamics and guide culturally tailored prevention efforts.

Accordingly, the present study hypothesized that individuals with higher conformity and social desirability tendencies will cluster together and exhibit distinct patterns of licit substance use and problematic use. Therefore, this study aimed to identify distinct participant profiles based on social desirability and conformity tendencies using cluster analysis. It also sought to examine the bivariate associations of these clusters with licit tobacco and alcohol use and problematic substance use in a multicultural context.

## 2 Methods

### 2.1 Study design and participants

A cross-sectional study with 460 participants was carried out between February and March 2025. A self-report online survey administered using Google® Forms was used to collect the data. Before accessing the questionnaire, participants were presented with a brief introductory statement that described the study purpose, the voluntary nature of participation, the eligibility criteria, and assurances of anonymity and confidentiality. The statement also specified that the study was conducted by researchers from Lebanese universities and had obtained ethical approval from the Ethics and Research Committee of the Lebanese International University.

The survey was distributed using a snowball sampling technique, a non-probability method in which an initial group of participants recruits further participants from within their own social networks, generating a progressively expanding sample. Specifically, a group of 10 communication liaisons, who collaborated directly with the research team, started disseminating the questionnaire among students and colleagues. These liaisons were personally known to the research team. They were selected based on their social networks spanning different demographic backgrounds (including age, sex, and geographic regions across Lebanon's eight governorates) and diverse occupational and educational settings to maximize the heterogeneity of the recruited sample. The survey link was disseminated primarily through social media platforms, including WhatsApp and Instagram, via private messages and group communications within these networks. Recipients were encouraged to further share the survey link with eligible individuals within their contacts.

This closed-network dissemination strategy reduces the likelihood of bot infiltration compared to open-link or crowdsourced surveys, as access was propagated through personal social contacts rather than being publicly posted. The inclusion criteria were as follows: adults aged 18 years and older, living in Lebanon. There was no financial compensation for participating in the study, and participation was completely voluntary. The dataset was additionally screened for implausible response patterns, including straight-lining (identical responses across all items) and logically contradictory answers, and any such responses were removed before analysis.

### 2.2 Ethical consideration

The study protocol was approved by the Ethics and Research Committee of Lebanese International University on February 3, 2025 (approval number: 2025ERC-010-LIUSOP). Prior to enrollment, all participants' informed consent was electronically obtained. All procedures were carried out in accordance with regulatory standards and ethical guidelines.

### 2.3 Sample size calculation

The minimum sample size was calculated using the G*Power software 3.1.9.7 for Windows (Heinrich Heine, Universität Düsseldorf, Düsseldorf, Germany). As multivariable regressions would be used to assess their correlates of dependent variables, and assuming a calculated effect size is f^2^ = 0.05 (small effect size) and expecting a squared multiple correlation of 0.05 (R^2^ deviation from 0) related to the Omnibus test of the regression, the minimum necessary sample will be *n* = 454, considering an alpha error of 5%, a power of 80%, and allowing 25 predictors to be included in the model. A total of 460 participants were included in the study.

### 2.4 Questionnaire

The online survey was available in English and Arabic (bilingual). The survey required approximately 10–15 min to be completed and was divided into two sections (Supplementary file 1).

The first section of the questionnaire gathered sociodemographic and behavioral data, including age, gender, marital and employment status, education, income, nationality, residency, and living area across Lebanon's eight governorates. Participants were also asked about substance use behaviors—current smoking (cigarette or waterpipe), alcohol consumption, and caffeine use while smoking—as well as whether their religious beliefs prohibit alcohol consumption. This section further assessed substance use and related problematic use, collecting detailed information on cigarette, waterpipe, and alcohol use; for cigarette smoking, data were obtained on smoking status, the number of cigarettes smoked per day, and the age at first cigarette. Waterpipe use was assessed by asking participants about their waterpipe smoking status, the number of nargilehs smoked per week, and the age at first waterpipe use. Alcohol consumption was evaluated by asking participants about their drinking status and the number of alcoholic drinks consumed per occasion.

The second section included five validated scales as follows:

### 2.5 The lebanese social desirability scale (LSDS)

The LSDS scale was created and developed in Lebanon to assess how much people tend to present themselves in a way that is socially acceptable. It consists of 36 items loaded on three factors: Virtuous Perfectionism (Factor 1), Independent Thinking (Factor 2), and socially Strategic Pragmatism (Factor 3). Factor 1 included 15 items with higher score represented perfectionist, virtuous, and impression management. Example items for factor 1: “I have never damaged a library book or store merchandise without reporting it”; “I seek to be myself rather than to follow others”; “I always declare everything at customs”. Factor 2 included 8 items, and a higher score was related to lower conscientiousness, lower impression management, and possibly higher openness to experience. Example items for factor 2: “When I was young, I sometimes stole things”; “I have some pretty awful habits”; “It would be hard for me to break any of my bad habits”. As for Factor 3 (13 items), a higher score was about overall realistic, socially adaptive, lower honesty, and moderately self-serving tendencies. Example items for factor 3: “Sometimes, I only help because I expect something in return”; “I sometimes think when people have a misfortune they only got what they deserved”; “I make a lot of effort to live up to what friends expect”. Each factor's score was calculated by adding the items where higher scores suggest more social desirability. Further information about this scale is presented in another publication (submitted paper) (Hajj et al., 2026). In the current manuscript, the factor 2 was inverted for clearer results (lower independent thinking with higher scores). The Cronbach's α in this sample were as follows: Virtuous Perfectionism = 0.945, Independent Thinking = 0.747, and Socially Strategic Pragmatism = 0.856. The structural validation of the scale is provided in the supplementary material (Supplement 2).

### 2.6 Conformity scale

The Conformity Scale, developed by Mehrabian and Stefl (Mehrabian & Stefl, 1995), was designed to assess traits related to conformity in the context of their study on loneliness, shyness, and conformity. The 11-item scale includes characteristics typical of conforming individuals, such as imitating dominant figures, following group norms, relying on others' advice, and being easily influenced. Example of items used: “I often rely on and act upon the advice of others”; “I am more independent than conforming in my ways” and “I prefer to find my own way in life rather than find a group I can follow”. Each item is rated on a 7-point scale ranging from 1 (“Not at all true of me”) to 7 (“Extremely true of me”). The total conformity score was computed as the mean of all items, with higher scores indicating greater conformity. The Cronbach's α in this sample was 0.925.

### 2.7 The lebanese cigarette dependence scale (LCD)

Severity of cigarette problematic use was assessed using the Lebanese Cigarette Dependence Scale (LCD) (Salameh et al., 2014), a validated instrument developed and standardized in the Lebanese population. The LCD evaluates the degree of nicotine problematic use across multiple dimensions, including compulsive use, craving, and loss of control. Example of items used: “I smoke for conviviality,” “How soon after you wake up do you smoke your first cigarette,” and “Do you smoke if you are so ill and bedridden.” Total scores reflect a continuum of problematic use severity, with higher scores indicating greater problematic use of cigarettes. The Cronbach's α in this sample was 0.935.

### 2.8 The lebanese waterpipe dependence scale (LWDS)

Waterpipe problematic use severity was measured using the Lebanese Waterpipe Dependence Scale (LWDS-11), a validated 11-item instrument specifically developed to assess problematic use in waterpipe smokers (Bahelah et al., 2017; Salameh et al., 2014). The scale captures key features of waterpipe problematic use, such as craving, loss of control, tolerance, and withdrawal-like symptoms. Example of items used: “Number of waterpipes you usually smoke per week,” “Number of times you could stop waterpipe for more than 7 days,” and “You smoke waterpipe to relax your nerves.” Items are rated on a Likert-type scale, and the total score reflects the overall severity of waterpipe problematic use, with higher scores indicating a more severe problematic use. The scale has demonstrated good psychometric properties across Lebanese and regional samples (Pahlavanzadeh et al., 2020). The Cronbach's α in this sample was 0.910.

### 2.9 Alcohol use disorders identification test (AUDIT)

Problematic alcohol use was assessed using the Alcohol Use Disorders Identification Test (AUDIT), a 10-item screening tool developed by the World Health Organization to identify hazardous and harmful alcohol consumption, as well as possible alcohol use disorder (Babor et al., 2001). Items cover frequency of drinking, quantity consumed, and alcohol-related consequences. Each item is scored from 0 to 4, yielding a total score ranging from 0 to 40. Example of items used: “How often do you have a drink containing alcohol,” “How often during the last year have you found that you were not able to stop drinking once you had started,” and “Have you or somebody else been injured as a result of your drinking.” Scores of 8–15 indicate hazardous use, scores of 16–19 indicate harmful use, and scores of 20 or above suggest possible alcohol use disorder. The AUDIT has been widely validated across diverse cultural and clinical populations and has demonstrated good reliability and validity in Middle Eastern samples. The Cronbach's α in this sample was 0.940.

### 2.10 Statistical analysis

The SPSS software version 25 was used to perform the data analysis. A descriptive analysis was performed, where categorical variables were expressed as absolute frequencies and percentages and quantitative variables as means and standard deviations. The distribution of normality was checked using visual inspection of the histogram, while the skewness and kurtosis were lower than 1. The results showed that the data were not normally distributed.

To classify the profile of the participants, a cluster analysis was done. Cluster analysis is a data-driven, distance-based method that groups individuals according to similarity across continuous variables, offering flexibility in handling multiple psychological constructs simultaneously. Given that our primary aim was exploratory to identify meaningful psychosocial profiles, cluster analysis was deemed the most appropriate approach. A two-step cluster analysis was conducted using the three social desirability factors (LSDS_Factor1, LSDS_Factor2, LSDS_Factor3) to identify homogeneous subgroups within the sample (Charron et al., 2023; Panlilio et al., 2020). In the first step, cases were pre-clustered, and multiple cluster solutions (k = 2, 3, and 4) were evaluated using both cohesion–separation indices and model fit criteria. Cluster adequacy was primarily assessed using the silhouette coefficient, which reflects the degree of within-cluster homogeneity relative to between-cluster separation, alongside the Bayesian Information Criterion (BIC), which evaluates model fit while penalizing model complexity. Additionally, the elbow method was applied to support the choice of the selected cluster solution, while the Gaussian mixture method was run as a sensitivity analysis related to the expected non-sphericity of data (Supplement 3).

The K-means clustering method was then performed to classify participants based on their scores on the conformity scale and the social desirability components, further confirming the three-cluster structure, with 10 random starts to reduce the risk of local minima. ANOVA was then applied to confirm that the clusters significantly differed on the variables used for clustering. The choice of these variables aligns with the study objective, which is to identify participant profiles based on social desirability and conformity tendencies and to examine their associations with tobacco and alcohol use and problematic use.

After that, a bivariate analysis was performed: the chi-square test was used to evaluate associations between the clusters and sociodemographic and behavioral characteristics; the Kruskal-Wallis test was used to compare the means of the scales used between the groups (the three clusters). A *p*-value less than 0.05 was considered significant.

Three separate generalized linear models were conducted, with problematic use of tobacco and alcohol as outcome variables (cigarette, waterpipe, and alcohol), while sociodemographic characteristics, religious beliefs, and cluster membership were independent variables. Estimates were calculated from the GENLIN models using Wald 95% confidence intervals, using a Gamma distribution with log link (to account for normality violation), and unstandardized coefficients (B) were reported. In addition, a fourth model with any problematic substance use as a dichotomous outcome variable was conducted: a Binomial distribution with logit link was used, and adjusted odds ratios (ORa; exponentiated coefficients) were reported. The substance use variable was also created in this study to assess cigarette, waterpipe, and alcohol use. Participants were asked the following items: “Are you a cigarette smoker,” “Are you a nargileh/waterpipe smoker,” and “Do you drink alcohol.” Participants reporting any use of these substances were classified as engaging in substance use.

## 3 Results

### 3.1 Sample description

The sociodemographic details of the 460 participants are shown in Table 1. They were mostly Lebanese residents, living in urban areas, females, single, with a university degree, unemployed, having little to no monthly income, and half of them living in Beirut and Mount Lebanon. Participants' mean age was 25 years.

**Table 1:** Sociodemographic characteristics of the participants (N = 460).

	N (%)
Gender	
Male	134 (29.1%)
Female	323 (70.2%)
I prefer not to answer	1 (0.2%)
Other	2 (0.4%)
Marital status	
Single / widowed / divorced	375 (81.5%)
Married	85 (18.5%)
Education level	
School level	29 (6.3%)
University level	431 (93.7%)
Nationality	
Lebanese	437 (95.0%)
Non-Lebanese	23 (5.0%)
Residency status	
Resident	453 (98.5%)
visitor	7 (1.5%)
Governorate	
Beirut	115 (25.0%)
Mount Lebanon	113 (24.6%)
North	56 (12.2%)
Akkar	26 (5.7%)
South	71 (15.4%)
Nabatieh	29 (6.3%)
Beqaa	40 (8.7%)
Baalbek	10 (2.2%)
Living place	
Urban	285 (62.0%)
Rural	175 (38.0%)
Monthly income	
No income	216 (47.0%)
Low	107 (23.3%)
Intermediate	108 (23.5%)
High	29 (6.3%)
Occupation	
Unemployed	357 (77.6%)
Employed	103 (22.4%)
Mean ± SD	
Age	25.35 ± 9.17

Cigarette smoking was reported by one third of the sample. The LCD scale scores for the sample had a mean of 5; the average number of cigarettes consumed per day was 13, and the mean age at smoking initiation was 17 years. Waterpipe smoking was reported by a third of participants, and the LWDS–11 mean score was 3. Among waterpipe users, the average consumption was 4 sessions per week, and the mean age at initiation was 18 years. Regarding alcohol consumption, one fifth of participants reported some use, and the AUDIT scores had a mean of 1.6. Among drinkers, the mean number of alcoholic drinks consumed per occasion was 2.5 (Table 2).

**Table 2:** Description of tobacco and alcohol use and associated scale scores.

Substance Use	Category	Frequency (%)
Cigarette smoker	No	372 (80.9%)
	Yes, previous smoker	21 (4.6%)
	Yes, current occasional smoker	28 (6.1%)
	Yes, current regular smoker	39 (8.5%)
LCD scale (Mean ± SD)		4.99 ± 8.49
Number of cigarettes smoked per day (Mean ± SD)		13.08 ± 10.19
Age at first cigarette (Mean ± SD)		16.79 ± 4.18
Waterpipe smoker	No	309 (67.2%)
	Yes, previous smoker	25 (5.4%)
	Yes, current occasional smoker	88 (19.1%)
	Yes, current regular smoker	38 (8.3%)
LWDS – 11 scale (Mean ± SD)		3.12 ± 5.60
Number of waterpipes smoked per week (Mean ± SD)		4.09 ± 3.94
Age at first waterpipe (Mean ± SD)		18.16 ± 3.94
Alcohol consumption	No	374 (81.3%)
	Yes, previous consumer	9 (2.0%)
	Yes, current regular consumer	17 (3.7%)
	Yes, occasional consumer	60 (13.0%)
AUDIT scale (Mean ± SD)		1.56 ± 4.69
Number of alcoholic drinks per occasion (Mean ± SD)		2.47 ± 2.49

Note: LCD: Lebanese Cigarette Dependence scale; LWDS: Lebanon Waterpipe Dependence Scale; AUDIT: Alcohol Use Disorders Identification.

### 3.2 Cluster analysis and bivariate comparisons of sociodemographic and behavioral variables

These analyses are reported in the Supplementary Material 3 (Table S1 and Supplementary Fig. S1), indicating that the three-cluster solution provides a reasonable separation among participant profiles, with stable and meaningful clusters. The 3-cluster solution demonstrated the highest silhouette coefficient (0.4), indicating the most optimal balance between cohesion and separation among the tested solutions. Although the BIC continued to decrease with increasing cluster number, reaching its lowest value in the 4-cluster solution, this improvement in fit was accompanied by a reduction in silhouette quality, suggesting potential over-extraction and reduced interpretability. In line with recommendations prioritizing both statistical adequacy and parsimony, the 3-cluster solution was therefore retained as the most appropriate representation of the data structure.

The K-means cluster analysis based on these variables identified three profiles: Cluster 1 (*N* = 127; 29.2%) showed the highest scores on virtuous perfectionism and conformity, suggesting individuals who strongly internalize social norms and strive to present themselves in a morally ideal manner; therefore, this cluster was labeled *Norm-Internalizing Perfectionists.* Cluster 2 (*N* = 143; 32.9%) demonstrated lower conformity and lower social desirability scores, reflecting greater independence from social expectations and norms, which led to the label *Detached Autonomists* and Cluster 3 (*N* = 165; 37.9%) combined relatively high conformity with higher scores on strategic pragmatism, indicating individuals who tend to adapt their behaviors strategically to social contexts while maintaining conformity, and was therefore labeled *Strategic Conformists* (Table 3).

**Table 3:** Participant profiles based on cluster analysis: distribution of psychological traits.

	Cluster 1	Cluster 2	Cluster 3	F-values descriptive	
	N = 127 (29.2%)	N = 143 (32.9%)	N = 165 (37.9%)		*P* value
Conformity scale	3.50	3.48	3.92	43.90	<0.001
LSDS1 (Virtuous Perfectionism)	74.35	29.94	53.98	1357.81	<0.001
LSDS2 (Independent Thinking - Reversed)	27.98	19.78	30.87	105.01	<0.001
LSDS3 (Socially Strategic Pragmatism)	29.71	26.28	37.38	115.80	<0.001

*Note:* Cluster names: Norm-Internalizing Perfectionists (Cluster 1); Detached Autonomists (Cluster 2); and Strategic Conformists (Cluster 3).

In the second step, the final 3-cluster solution was evaluated for internal validity using one-way analyses of variance (ANOVAs). Results indicated that all three social desirability factors significantly differentiated the clusters, with large F-values observed for LSDS_Factor1 (F = 604.57, *p* < .001), LSDS_Factor2 (F = 282.35, p < .001), and LSDS_Factor3 (F = 251.35, p < .001). These findings confirm strong between-cluster heterogeneity and support the validity and robustness of the selected cluster solution.

The bivariate analysis further revealed significant associations with sociodemographic and behavioral factors: Cluster 2 included older participants with higher smoking and drinking rates, while Cluster 1 had the most university graduates, strong religious adherence, and the highest virtuous perfectionism scores, and Cluster 3 showed the highest conformity, non-independent thinking, and pragmatism (Table 3, Table 4). No significant differences in gender distribution and waterpipe smoking were found. As for problematic substance use, significantly higher misuse was found for cluster 2 (Detached autonomists), followed by cluster 3 (Strategic conformists), and finally cluster 1 (Norm internalizing perfectionists) (p < .001) (Table 4).

**Table 4:** Bivariate Associations Between Cluster Membership and Sociodemographic and Behavioral Variables.

	Cluster 1*	Cluster 2*	Cluster 3*	P value
	N (%)	N (%)	N (%)	
Gender				
Male	52 (26.4%)	31 (27.9%)	44 (34.6%)	0.292
Female	143 (72.6%)	79 (71.2%)	83 (65.4%)	
Education level				
School level	2 (1.2%)	14 (11%)	13 (9.6%)	<0.001
University level	168 (98.8%)	113 (89%)	122 (93.3%)	
Religious beliefs forbid alcohol				
No	19 (11.2%)	29 (22.8%)	32 (23.7%)	0.007
Yes	151 (88.8%)	98 (77.2%)	103 (76.3%)	
Current cigarette smoker				
No	162 (95.3%)	89 (70.1%)	121 (89.6%)	<0.001
Yes	8 (4.7%)	38 (29.9%)	14 (10.4%)	
Current waterpipe smoker				
No	124 (72.9%)	95 (74.8%)	95 (70.4%)	0.720
Yes	46 (27.1%)	32 (25.2%)	40 (29.6%)	
Current alcohol consumer				
No	157 (92.4%)	103 (81.1%)	372 (86.1%)	0.009
Yes	13 (7.6%)	24 (18.9%)	23 (17%)	
Consume alcohol while smoking				
No	166 (97.6%)	114 (89.8%)	125 (92.6%)	0.017
Yes	4 (2.4%)	13 (10.2%)	10 (7.4%)	
Consume caffeine while smoking				
No	140 (82.4%)	72 (56.7%)	93 (68.9%)	<0.001
Yes	30 (17.6%)	55 (43.3%)	42 (31.1%)	
	Mean (SD)	Mean (SD)	Mean (SD)	P value
Age (years)	22.79 (5.22)	28.01 (11.88)	24.28 (7.92)	<0.001
LCD	3.55(7.21)	8.59(10.58)	4.09(7.42)	<0.001
LWDS	1.70(3.69)	5.55(7.25)	2.69(5.15)	<0.001
AUDIT	0.65(2.22)	3.62(7.52)	0.86(3.47)	<0.001

*Note:* Comparisons were obtained using chi-square tests for categorical variables and one-way ANOVA for continuous variables.

Cluster names: Norm-Internalizing Perfectionists (Cluster 1); Detached Autonomists (Cluster 2); and Strategic Conformists (Cluster 3).

### 3.3 Multivariable analysis of problematic cigarette use

After adjustment for all covariates (including religion), occupation, gender, education, and cluster membership were significantly associated with LCD. Being employed was associated with higher LCD. Female gender and university education were associated with lower LCD. As for clusters, Detached Autonomists showed higher LCD, whereas Strategic Conformists did not differ from the Norm Internalizing Perfectionists. Marital status, age, or religious prohibition of alcohol were not associated with LCD (Table 5; Model 1).

**Table 5:** Multivariable analyses of Licit Substance Problematic Use.

Model 1 – Cigarette Problematic Use (LCD): Generalized Linear Model; Gamma with Log Link				
Correlate	Details	B	p-value	95% CI
Marital status	Married vs Single/widowed/divorced (ref)	−0.033	0.861	−0.396 to 0.331
Occupation	Employed vs Unemployed (ref)	0.340	0.018	0.057 to 0.622
Gender	Females vs Males (ref)	−0.662	<0.001	−0.900 to −0.424
Education	University vs School (ref)	−0.702	0.003	−1.171 to −0.233
Cluster	Strategic Conformists vs Norm Internalizing Perfectionists (ref)	−0.191	0.123	−0.435 to 0.052
	Detached autonomists vs Norm Internalizing Perfectionists (ref)	0.622	<0.001	0.360 to 0.884
Age	Per 1-year increase	0.012	0.155	−0.005 to 0.029
SES-C scale	Per 1-unit increase	−0.033	0.086	−0.071 to 0.005
Religious prohibition of alcohol	Yes vs No (ref)	0.133	0.331	−0.135 to 0.401
Model 2 – Waterpipe Problematic Use (LWDS): Generalized Linear Model; Gamma with Log Link				
Correlate	Details	B	p-value	95% CI
Marital status	Married vs Single/widowed/divorced (ref)	0.090	0.598	−0.245 to 0.425
Occupation	Employed vs Unemployed (ref)	0.013	0.927	−0.265 to 0.291
Gender	Females vs Males (ref)	−0.058	0.609	−0.281 to 0.165
Education	University vs School (ref)	−0.114	0.607	−0.548 to 0.321
Cluster	Strategic Conformists vs Norm Internalizing Perfectionists (ref)	−0.470	<0.001	−0.703 to −0.236
	Detached autonomists vs Norm Internalizing Perfectionists (ref)	0.478	<0.001	0.227 to 0.729
Age	Per 1-year increase	0.003	0.698	−0.014 to 0.021
SES-C scale	Per 1-unit increase	−0.031	0.106	−0.069 to 0.007
Religious prohibition of alcohol	Yes vs No (ref)	−0.069	0.613	−0.335 to 0.198
Model 3 – Alcohol Problematic Use (AUDIT): Generalized Linear Model; Gamma with Log Link				
Correlate	Details	B	p-value	95% CI
Marital status	Married vs Single/widowed/divorced (ref)	0.219	0.145	−0.076 to 0.513
Occupation	Employed vs Unemployed (ref)	−0.268	0.033	−0.515 to −0.021
Gender	Females vs Males (ref)	−0.055	0.586	−0.254 to 0.144
Education	University vs School (ref)	−0.180	0.351	−0.560 to 0.199
Cluster	Strategic Conformists vs Norm Internalizing Perfectionists (ref)	−0.174	0.097	−0.379 to 0.031
	Detached autonomists vs Norm Internalizing Perfectionists (ref)	0.776	<0.001	0.560 to 0.992
Age	Per 1-year increase	−0.011	0.135	−0.025 to 0.003
SES-C scale	Per 1-unit increase	−0.040	0.014	−0.072 to −0.008
Religious prohibition of alcohol	Yes vs No (ref)	−0.573	<0.001	−0.813 to −0.332
Model 4 – Any Substance Problematic Use: Logistic regression				
Correlate	Details	ORa	p-value	95% CI
Marital status	Married vs Single/widowed/divorced (ref)	0.532	0.116	0.242 to 1.168
Occupation	Employed vs Unemployed (ref)	1.048	0.885	0.555 to 1.982
Gender	Females vs Males (ref)	0.596	0.042	0.362 to 0.982
Education	University vs School (ref)	0.365	0.152	0.092 to 1.448
Cluster	Strategic Conformists vs Norm Internalizing Perfectionists (ref)	0.811	0.408	0.494 to 1.331
	Detached autonomists vs Norm Internalizing Perfectionists (ref)	2.963	<0.001	1.719 to 5.107
Age	Per 1-year increase	1.092	0.002	1.033 to 1.155
SES-C scale	Per 1-unit increase	1.000	0.996	0.923 to 1.082
Religious prohibition of alcohol	Yes vs No (ref)	0.315	<0.001	0.167 to 0.595

Note: LCD: Lebanese Cigarette Dependence scale; LWDS: Lebanon Waterpipe Dependence Scale; AUDIT: Alcohol Use Disorders Identification Test; SES—C: Socioeconomic Status Composite scale.

### 3.4 Multivariable analysis of problematic waterpipe use

In this model, the only significant predictors were cluster membership. Relative to Norm Internalizing Perfectionists, Strategic Conformists had lower LWDS, while Detached Autonomists had higher LWDS. All other variables were not significantly associated with LWDS (Table 5; Model 2).

### 3.5 Multivariable analysis of problematic use of alcohol

Several covariates were significantly associated with AUDIT. Detached autonomists had higher AUDIT scores versus Norm Internalizing Perfectionists, while Strategic Conformists showed a non-significant trend toward lower values. Importantly, religious prohibition of alcohol was associated with substantially lower AUDIT. Working and a higher socioeconomic status were associated with lower AUDIT scores. The remaining variables were not significant: marital status, gender, education, and age (Table 5; Model 3).

### 3.6 Multivariable analysis of moderate/severe problematic use of any licit substance

In the fully adjusted logistic model, significant predictors were gender, age, cluster membership, and religious prohibition of alcohol. Relative to Norm Internalizing Perfectionists, Detached Autonomists had markedly higher odds, while Strategic Conformists did not differ. Female gender and religious prohibition of alcohol were associated with substantially lower odds of problematic substance use. Non-significant variables included marital status, occupation, education, and socioeconomic status.

## 4 Discussion

The study reveals that religiosity, gender, age, education, and conformity profile jointly shape substance use patterns among Lebanese young adults. Three profiles emerged, highlighting how personality and social norms influence risk behaviors: Norm-Internalizing Perfectionists (Cluster 1); Detached Autonomists (Cluster 2); and Strategic Conformists (Cluster 3).

### 4.1 Conformity, social desirability, and mechanisms shaping substance use

Social desirability strongly predicted lower self-reported substance use, highlighting potential under-reporting, especially in Cluster 1 (Norm-Internalizing Perfectionists), whose high conformity and perfectionism aligned with protective cultural and religious values. These individuals' consistent low substance engagement suggests they may benefit from norm-based health promotion and cognitive-behavioral interventions targeting perfectionism and adaptive coping (Egan, Wade, & Shafran, 2011). Cluster 2 (Detached autonomists) showed the highest risk behaviors and poly-substance use, indicating a need for harm-reduction strategies addressing their autonomous attributes. Cluster 3 (Strategic conformists) displayed intermediate levels of problematic substance use, supporting interventions that reinforce pragmatic, autonomy-based coping while respecting norm conformity.

More specifically, problematic cigarette use was shaped primarily by a mix of socio-structural positioning (employment/education) (Hiscock, Bauld, Amos, Fidler, & Munafò, 2012) and social desirability profile (Singh et al., 2022), with gender differences potentially reflecting unmeasured contextual exposures, coping styles, or reporting/measurement differences not captured by SES or religion (Garrett, Martell, Caraballo, & King, 2019). As for waterpipe, the effects of cluster membership without other sociodemographic factors are consistent with problematic waterpipe use being more tightly coupled to underlying psychosocial/behavioral phenotypes than to conventional sociodemographic gradients (Berg et al., 2020); null associations for SES, education, and religion may reflect mediation via cluster-related traits (VanderWeele, 2016), limited variability, or outcome-specific mechanisms (Greenland, Pearl, & Robins, 1999). Collectively, the correlates of alcohol problematic use are consistent with risk being shaped by psychosocial phenotype (detached autonomous cluster) (Kotov et al., 2017), structural resources (SES), and well-known culturally or religiously grounded norms or constraints on drinking (Alhashimi et al., 2018; Collins, 2016); the employment association may reflect differential social exposure and contextual structures for alcohol use (Frone, 2016) or residual confounding by work-related context.

### 4.2 Sociodemographic characteristics' associations with problematic substance use

After adjusting for cluster profiles, the analysis revealed clear gender-based patterns. Male participants showed the highest risk of problematic licit substance use, mostly associated with rejection of traditional norms, likely driven by masculinity pressures and emerging adulthood stressors. This aligns with evidence that males in traditional societies often face greater pressure to engage in substance use as an expression of masculinity (Ja'afar et al., 2025; Yang et al., 2008). In contrast, females showed lower problematic use, mostly protected by their high conformity and perfectionism. These gender differences reflect broader Middle Eastern cultural patterns where women face greater social sanctions for substance use, leading to either complete abstinence or highly secretive use patterns (Jaber et al., 2015; Khoshnood et al., 2022). The elevated risk among males indicates the need for prevention programs targeting masculine social pressures and promoting healthier coping and social integration strategies.

Age also emerged as a significant differentiating factor between clusters, with relatively older age specifically characterizing Cluster 2's high-risk profile. Moreover, older individuals presented higher odds of any problematic substance use; their increased life experience and autonomy appear to facilitate risk-taking behaviors as they navigate occupational stressors and evolving social networks (Ibrayeva et al., 2025; Mankelkl & Kinfe, 2025). Moreover, this finding aligns with evidence that older individuals may accumulate more diverse substance-use experiences over time, potentially reflecting greater exposure to different social settings, substance-use opportunities, and reinforcement processes (Schulenberg et al., 2018).

Cluster 1 (norm internalizing perfectionists) included the highest percentage of participants with religious alcohol prohibition and the lowest alcohol consumption percentage, in line with traditional assumptions that link high conformity to lower risk-taking (Esteban-Gonzalo et al., 2020). In the same line, the cluster 2 (detached autonomists) behavior reflects a detachment from traditional norms, possibly viewing substance use as an acceptable stress-coping practice despite religious prohibitions (Bizri, El Hayek, Beaini, Kobeissy, & Talih, 2021; Goh et al., 2024). This situation reflects the Lebanese multi-religious context, 77.2–88.8% reported alcohol-prohibiting beliefs, reflecting enduring Islamic and conservative Christian influences (Bobo & Husten, 2000; Karam et al., 2000). These norms can protect against substance use but also complicate prevention and treatment efforts due to stigma in reporting and help-seeking (Mallik, Starrels, Shannon, Edwards, & Nahvi, 2021).

Education showed clear differences across clusters, with Cluster 1 most strongly linked to higher education. Nevertheless, educated participants showed overall similar problematic use of licit substances versus school-educated participants, except for problematic cigarette use. The latter is explained by education-driven rational decision-making about cigarette use risks rather than conforming to peer pressures (Brady & Randall, 1999), and suggests that intellectual independence and critical thinking may protect against peer and societal pressures (Conrod et al., 2025). Furthermore, their pragmatic approach to life may involve critical evaluation of the risks associated with substance use, consistent with research showing that higher education correlates with lower substance use severity (Bugbee, Beck, Fryer, & Arria, 2019). Similarly, Cluster 1's uniformly high education levels (98.8% university-educated) combined with their strong conformity created a unique profile where academic achievement and social compliance reinforced each other in maintaining the most restrictive substance use patterns observed in the study, reflecting a growing trend of individualism and resistance to societal pressure, especially among educated women in post-crisis Lebanon (Iwamoto & Mui, 2020). However, this is not the case for waterpipe and alcohol, suggesting different dynamics when compared to cigarettes: cigarette, waterpipe, and alcohol use each reflect unique social contexts, with waterpipe smoking more strongly tied to conformity and viewed as more socially acceptable than cigarettes in Middle Eastern culture (Bahelah et al., 2017; Darijani et al., 2025; Pahlavanzadeh et al., 2020), while alcohol acceptability is strongly linked to religious beliefs, as previously explained. This complexity warrants further investigation in future studies, along with its potential explanations, namely differential social pressure or mediation through social desirability and conformity aspects.

### 4.3 Public health implications

The identification of distinct psychosocial profiles also has potential implications for public health communication and prevention strategies. Rather than applying universal approaches, substance use interventions in Lebanon may be more effective when tailored to specific cluster profiles, accounting for cultural norms, conformity pressures, and individual behavioral tendencies. The protective role of religious prohibition further suggests that culturally and religiously informed messaging could reinforce norms discouraging substance use. Additionally, gender, age, and education differences highlight the need for interventions sensitive to varying risk factors and levels of health literacy across subgroups. Overall, these findings support the development of cluster-tailored public health campaigns, school-based programs, and workplace initiatives that allocate resources more efficiently and are better suited to the psychosocial realities of the Lebanese population.

### 4.4 Limitations and future directions

Despite its valuable findings, this study has several limitations affecting generalizability. The sample was largely homogeneous, including young, female, urban, and mostly university-educated (93.7%) and unemployed (77.6%), limiting applicability beyond this group, though enhancing internal consistency. The cross-sectional design also prevents causal inference, highlighting the need for longitudinal studies to track how cluster profiles and their association with problematic substance use might evolve (Norm-Internalizing Perfectionists, Detached Autonomists, and Strategic Conformists). Online snowball sampling, while appropriate given the absence of a defined sampling frame, further limits representativeness due to its non-random nature. The sample was characterized by a high proportion of university-educated participants, which does not fully reflect the educational distribution of the Lebanese population. This pattern likely reflects the recruitment method, as online snowball sampling tends to reach younger and more digitally connected individuals, including university students and recent graduates. In the present study, the combination of high education levels and high unemployment rates among participants may reflect the broader economic situation in Lebanon, where many young graduates face limited employment opportunities. Self-reported data may further introduce reporting bias, particularly underreporting of substance use due to cultural sensitivities; however, anonymity was ensured, and social desirability was directly measured via the LSDS and incorporated into the analytical framework. Administering the survey in both English and Arabic, though necessary to enhance comprehension, may have introduced minor variability in question interpretation.

This study did not assess lifetime substance use, as the questionnaire focused on current and past use within defined categories.

Finally, the use of cluster analysis, while appropriate for exploratory purposes, lacks the probabilistic framework and formal fit indices of latent class analysis (LCA), which may limit inferential robustness (Oberski, 2016, Spurk et al., 2020. Moreover, due to the expected non-sphericity of the psychological factors, we also run a Gaussian mixture model for sensitivity: comparison between the original clustering solution and the Gaussian mixture model allowing for full covariance structures showed moderate agreement (Adjusted Rand Index = 0.58), suggesting that the initial clusters capture meaningful structure and profiles, although they do not fully reflect the underlying anisotropic distribution of the data. Nevertheless, K-means cluster analysis remains widely used in behavioral and public health research for exploratory purposes and hypothesis generation.

Future research should include behavioral or biological validation and adopt multi-level approaches integrating biological, social, and psychological factors for a more comprehensive understanding of substance use clustering. Prospective studies with pathway analysis are suggested to clarify the complex interplay between the studied concepts and personal characteristics of the Lebanese population.

## 5 Conclusion

Considering social desirability and conformity allows for more precise identification of substance use clusters, guiding targeted prevention and intervention. In summary, problematic substance use risk was concentrated in the detached autonomous phenotype and increased with age, while religious prohibition appeared strongly protective, potentially via reduced opportunity, stronger social sanctions, and supportive community norms; the gender effect may reflect differential exposure patterns, social constraints, or gender-linked vulnerability pathways. This complex interplay of psychological and social-behavioral characteristics, cultural values, age, gender, and education highlights the need for culturally sensitive, gender-specific, and cluster-tailored public health and psychosocial interventions rather than universal approaches.

## CRediT authorship contribution statement

**Rana Hatem:** Writing – original draft. **Sandrella Bou Malhab:** Writing – review & editing, Formal analysis. **Chadia Haddad:** Writing – review & editing, Formal analysis. **Hala Sacre:** Writing – review & editing. **Aline Hajj:** Writing – review & editing. **Fouad Sakr:** Writing – review & editing. **Roy Eid:** Writing – review & editing. **Georgia Chkayban:** Writing – review & editing. **Clara Gerges:** Writing – review & editing. **David Karam:** Writing – review & editing. **Marguerite Ghostin:** Writing – review & editing. **Dalal Youssef:** Writing – review & editing. **Pascale Salameh:** Writing – review & editing, Validation, Supervision, Conceptualization.

## Funding

No funding was received for conducting this study.

## Declaration of competing interest

The authors declare that they have no known competing financial interests or personal relationships that could have appeared to influence the work reported in this paper.

## Data Availability

Data will be made available on request.

## Appendix A Supplementary data

Supplementary Material 1: Study Questionnaire

Supplementary material 2: Construct Validity and Reliability of the Lebanese Social Desirability Scale (LSDS)

Supplementary material 3: Cluster Properties and Normality Checking
