# Supplementary material for: Cluster analysis of conformity and social desirability: Association with use and problematic use of licit substances
Source: Addict Behav Rep. 2026 May 20;23:100710. doi: 10.1016/j.abrep.2026.100710 (PMC13223954; doi:10.1016/j.abrep.2026.100710)
Supplement: Supplementary file 1 — Supplementary Material 1: Study Questionnaire [file mmc1.docx]

**Social desirability, smoking, and alcohol consumption:**

**a cross-sectional study from Lebanon**

Dear participant,

You are invited to participate in this study conducted by a group of academic researchers to assess social desirability, smoking, and alcohol consumption in Lebanon.

All gathered information will be treated confidentially. Your participation in this study is voluntary. Completing the questionnaire requires 20 minutes and indicates your consent to participate.

Thank you in advance for your time and participation.

عزيزي المشارك ،

إذا كنت لبنانيا ، أنت مدعو للمشاركة في هذه الدراسة التي تجريها مجموعة من الباحثين الأكاديميين لتقييم الاستحسان الاجتماعي والتدخين واستهلاك الكحول في لبنان.

سيتم التعامل مع جميع المعلومات التي سيتم جمعها في هذه الدراسة بسرية تامة. مشاركتك في هذا البحث طوعية. إكمال الاستبيان يستغرق عشرين دقيقة ويشير إلى موافقتك على المشاركة.

شكراً على تعاونك.

**Please check all the below statements to proceed to the survey.**

يرجى اختيار جميع البيانات أدناه للمتابعة إلى الاستبيان.

☐ I have read and understood the above information

لقد قرأت وفهمت المعلومات المذكور أعلاه

☐ I understand that my participation is voluntary

أدرك أن مشاركتي في هذه الدراسة طوعية

☐ I understand that my data will be kept confidential

أدرك أن المعلومات التي سأقدمها ستبقى سرية

☐ I agree to participate in this study

أوافق على المشاركة في البحث المذكور أعلاه

# SOCIODEMOGRAPHIC AND OTHER CHARACTERISTICS

# الخصائص الاجتماعية والديموغرافية وغيرها

|  | **Age:** | العمر: |
| --- | --- | --- |
|  | **Gender:**   - - - 1. Male;       2. Female;       3. I prefer not to answer;       4. other | **الجنس:**  ذكر؛ انثى؛ أفضل عدم الإجابة؛ آخر |
|  | **Marital status:**   - - - 1. Single;       2. Married/ In a solid relationship;       3. Widowed;       4. Divorced;       5. unmarried | **الوضع الاجتماعي:**  أعزب (عزباء)؛ متزوج(ة)؛ أرمل(ة)؛ مطلق(ة)؛ في علاقة متينة، غير متزوج(ة) |
|  | **Nationality:**   - - - 1. Lebanese;       2. Non-Lebanese | **الجنسية:**  لبناني؛ غير لبناني |
|  | **Residency status in Lebanon:**   - - - 1. Resident;       2. Visitor | **وضع الإقامة في لبنان:**  زائر؛ مقيم |
|  | **Weight (in Kg):** | **الوزن (بالكيلو جرام):** |
|  | **Height (in cm):** | **الطول (سم):** |
|  | **Current governorate of living:**   - - - 1. Beirut;       2. Mount Lebanon;       3. North;       4. Akkar;       5. South;       6. Nabatieh;       7. Beqaa;       8. Baalbeck/Hermel | **محافظة السكن الحالية:**  بيروت؛ جبل لبنان؛ الشمال؛ عكار؛ الجنوب؛ النبطية؛ البقاع؛ بعلبك/الهرمل |
|  | **Living place status**:   - - - 1. Urban;       2. Rural | **وضع السكن الحالي:**  مدينة؛ ضيعة |
|  | **How do you describe the housing/ neighborhood condition?**   - - - 1. Very clean;       2. Relatively Clean;       3. Relatively dirty;       4. Very dirty | **كيف تصف حالة السكن / الحي؟**  نظيف جداً؛ نظيف نسبياً؛ متسخ نسبياً؛ متسخ جداً |
|  | **Number of persons living in the same household, including you:** | **عدد الأشخاص الذين يعيشون في نفس المنزل، بما فيهم أنت:** |
|  | **Number of dependent children (if none, put 0):** | **عدد الأطفال الذين على عاتقك (إذا لا أطفال، ضع صفر):** |
|  | **Number of rooms in your house, excluding the kitchen and bathrooms:** | **عدد الغرف في المنزل (عدا الحمام والمطبخ):** |
|  | **Your monthly income in US dollars:**   - - - 1. No income;       2. Low: below 500$;       3. Intermediate: 501-2000$;       4. High: over 2000$ | **دخلك الشهري الفردي بالدولار الأمريكي:**  لا مدخول؛ منخفض: أقل من 500$؛ متوسط: 501-2000$؛ مرتفع: أكثر من 2000$ |
|  | **Occupation:**   - - - 1. I do not work/I do not work currently;       2. Retired;       3. University student;       4. Self-employed;       5. Healthcare professional;       6. Managerial or administrative position;       7. Manual worker;       8. Public sector employee;       9. Private sector employee;       10. Military corps;       11. Other       12. Housewife; | **المهنة:**  لا اعمل/لا اعمل حالياً؛ متقاعد؛ ربة منزل؛ طالب جامعي؛ اعمل لحسابي الخاص؛ اعمل في الرعاية الصحية؛ اشغل منصب تنفيذي أو إداري؛ عامل يدوي؛ موظف في القطاع العام؛ موظف في القطاع الخاص؛ اعمل في السلك العسكري؛ غير ذلك |
|  | **Is there a member of your family who is a healthcare professional?**  Yes  No | **هل هناك فرد من عائلتك متخصص في المجال الصحي؟**  نعم لا |
|  | **Education level of the father:**   - - - 1. Illiterate or did not receive formal education;       2. School level;       3. University level | **المستوى التعليمي** **للوالد:**  أمي أو لم يتلق أي تعليم رسمي؛ مستوى مدرسي؛ مستوى جامعي |
|  | **Education level of the mother:**  Illiterate or did not receive formal education; School level; University level | **المستوى التعليمي** **للوالدة:**  أمي أو لم تتلق أي تعليم رسمي؛ مستوى مدرسي؛ مستوى جامعي |
|  | **Do your religious beliefs prohibit you from smoking cigarettes?** Yes No | **هل تمنعك معتقداتك الدينية من تدخين السيجارة؟** نعم لا |
|  | **Do your religious beliefs prohibit you from smoking waterpipes?** Yes No | **هل تمنعك معتقداتك الدينية من تدخين الارجيلة؟** نعم لا |
|  | **Do your religious beliefs prohibit you from smoking other tobacco products (cigar, cigarillo) or vaping?** Yes No | **هل تمنعك معتقداتك الدينية من تدخين منتجات التبغ الأخرى (السيجار والسيجاريلو) أو التدخين الإلكتروني؟** نعم لا |
|  | **Do your religious beliefs prohibit you from drinking alcohol?**  Yes No | **هل تمنعك معتقداتك الدينية تناول الكحول؟**  نعم لا |

# HEALTH-RELATED QUESTIONS

# أسئلة متعلقة بالصحة

|  | **How often do you exercise?**   - - - 1. Daily;       2. Weekly;       3. Occasionally;       4. Rarely;       5. Never | **كم مرة تمارس الرياضة؟**  يومياً؛ أسبوعياً؛ من حين لآخر؛ نادراً؛ أبداً |
| --- | --- | --- |
|  | **How often do you eat fast food?**  Daily; Weekly; Occasionally; Rarely; Never | **كم مرة تتناول الوجبات السريعة؟**  يومياً؛ أسبوعياً؛ من حين لآخر؛ نادراً؛ أبداً |
|  | **Do you have any chronic disease? (Yes/No):**  Arthritis  Asthma  Cancer  Chronic kidney disease  Chronic obstructive pulmonary disease (COPD)  Coronary artery disease  Crohn’s disease  Diabetes  Dyslipidemia  Epilepsy  Heart failure  Hypertension  Multiple sclerosis  Obesity  Schizophrenia  Thyroid disease  Ulcerative colitis  Depression  Anxiety  Other diseases | **هل أنت مصاب بأي مرض مزمن؟ (نعم / لا):**  **التهاب المفاصل**  **الربو**  **سرطان**  **فشل كلوي مزمن**  **مرض الانسداد الرئوي المزمن (COPD)**  **مرض القلب التاجي**  **مرض كرون**  **داء السكري**  **شحميات في الدم**  **الصرع**  **فشل في القلب**  **ارتفاع ضغط الدم**  **التصلب اللويحي المتعدد**  **بدانة**  **انفصام الشخصية**  **مرض الغدة الدرقية**  **التهاب القولون التقرحي**  **الاكتئاب**  **القلق**  **أمراض أخرى** |
|  | **Do you have easy access to healthcare?**  No; Yes | **هل لديك سهولة للوصول إلى الرعاية الصحية؟**  لا؛ نعم |
|  | **How often do you go for a general medical checkup?**   - - - 1. Yearly;       2. Every 2-3 years;       3. Only when necessary;       4. Never | **كم مرة تذهب لإجراء فحص طبي عام؟**  سنوياً؛ كل 2-3 سنوات؛ فقط عند الضرورة؛ أبداً |
|  | **What is your health coverage?**   - - - 1. Private insurance;       2. National Social Security Fund;       3. Ministry of Public Health;       4. Self-payer;       5. Public insurance (Army, COOP, Internal Security Forces);       6. Other insurance | **ما هي تغطيتك الصحية؟**  التأمين الخاص؛ الصندوق الوطني للضمان الاجتماعي؛ وزارة الصحة العامة؛ أدفع تكاليف العلاج بنفسي؛ تأمين مؤسسات عامة (الجيش، تعاونية موظفي الدولة، قوى الأمن الداخلي)؛ تأمين آخر |
|  | **Does any first-degree family member have cancer?**  No; Yes | **هل يعاني أي من أفراد أسرتك من الدرجة الأولى من السرطان؟**  لا؛ نعم |

# THE SOCIOECONOMIC STATUS COMPOSITE SCALE (SES-C)

# المقياس المركب للحالة الاجتماعية الاقتصادية

|  | **To which social class do you belong?**   - - - 1. Poor;       2. Lower-middle class;       3. Upper-middle class;       4. Wealthy;       5. I prefer not to answer | **إلى أي طبقة إجتماعية تنتمي؟**  فقيرة؛ الطبقة الوسطى الدنيا؛ الطبقة الوسطى العليا؛ ثرية؛ أفضل عدم الإجابة |
| --- | --- | --- |
|  | **Household monthly income in US dollars:**   - - - 1. No income;       2. Low: below 500$;       3. Intermediate: 501-2000$;       4. High: over 2000$ | **الدخل الشهري للأسرة بالدولار الأمريكي:**  لا مدخول؛ منخفض: أقل من 500$؛ متوسط: 501-2000$؛ مرتفع: أكثر من 2000$ |
|  | **The InCharge Financial Distress/Financial Well-Being (IFDFW) Scale***  1. What do you feel is the level of your financial stress today? (1= Overwhelming stress to 10= No stress at all)  2. How satisfied are you with your present financial situation? (1= Dissatisfied to 10= Satisfied)  3. How do you feel about your current financial situation? (1= I feel overwhelmed to 10= I feel comfortable)  4. How often do you worry about being able to meet normal monthly living expenses? (1= Worry all the time to 10= Never worry)  5. How confident are you that you could find the money to pay for a financial emergency that costs about 2000 USD? (1= No confidence to 10= High confidence)  6. How often does this happen to you? You want to go out to eat, go to a movie or do something else and don’t go because you can’t afford to? (1= All the time to 10= Never)  7. How frequently do you find yourself just getting by financially and living paycheck to paycheck? (1= All the time to 10= Never)  8. How stressed do you feel about your personal finances in general? (1= Overwhelming stress to 10= No stress at all) | **مقياس InCharge للضائقة المالية/الرفاهية المالية***  1. ما هو شعورك حول مستوى ضغطك المالي اليوم؟  (من 1= ضغط ساحق إلى 10= لا ضغط على الإطلاق)  2. ما مدى رضاك عن وضعك المالي الحالي؟  (من 1= غير راضٍ/ية على الإطلاق إلى 10= راضٍ/ية)  3. ما هو شعورك حول وضعك المالي الحالي؟  (من 1= أشعر بالعجز إلى 10= أشعر بالراحة)  4. كم مرة تقلق/ين بشأن قدرتك على تلبية نفقات المعيشة الشهرية العادية؟  (من 1= قلق طوال الوقت إلى 10= لا قلق أبداً)  5. ما مدى ثقتك في أنه يمكنك العثور على المال لدفع ثمن أي طارئ مالي قد يكلف حوالي 2000 دولار أمريكي؟  (من 1= لا ثقة إلى 10= ثقة عالية)  6. كم مرة يحدث هذا لك: تريد/ين الخروج لتناول الطعام أو الذهاب إلى السينما أو القيام بنشاط آخر ولا تذهب/ين لأنك لا تستطيع/ين دفع الثمن؟  (من 1= طوال الوقت إلى 10= أبداً)  7. كم مرة تجد/ين نفسك تحاول/ين أن تتدبر/ي أمرك مادياً لتعيش/ي بانتظار الأجر المقبل؟  (من 1= طوال الوقت إلى 10= أبداً)  8. كيف هو شعورك بالضغط تجاه ماليّتك الشخصية بشكل عام؟  (من 1= ضغط ساحق إلى 10= لا ضغط على الإطلاق) |
|  | **Are you in debt?**   1. No; 2. Yes; 3. I prefer not to answer | **هل انت مديون؟**  لا؛ نعم؛ أفضل عدم الإجابة |
|  | **Do you get financial help from others (e.g., family, friends, NGOs)?**   1. No; 2. Yes, regularly; 3. Yes, sometimes; 4. I prefer not to answer | **هل تحصل على مساعدة مالية من الآخرين (مثلاً، العائلة والأصدقاء والمنظمات غير الحكومية)؟**  لا؛ نعم بانتظام؛ نعم احياناً؛ أفضل عدم الإجابة |
|  | **Your education level:**  Illiterate or did not receive formal education; School level; University level | **مستواك التعليمي:**  أمي أو لم تتلق أي تعليم رسمي؛ مستوى مدرسي؛ مستوى جامعي |
|  | **Education level of the head of the family (including the participant):**  Illiterate or did not receive formal education; School level; University level | **المستوى التعليمي لرب العائلة (بما في ذلك المشارك):**  أمي أو لم يتلق أي تعليم رسمي؛ مستوى مدرسي؛ مستوى جامعي |
|  | **Work status of the participant:**   - - - 1. Working;  1. Not working | **وضع العمل للمشارك:**  يعمل؛ لا يعمل |
|  | **Work status of the head of the family:**  Working; Not working | **وضع العمل لرب العائلة:**  يعمل؛ لا يعمل |

# CONSUMPTION OF TOBACCO, ALCOHOL, AND CAFFEINE

# استهلاك التبغ والكحول والكافيين

|  | **Are you a cigarette smoker?**   1. No; 2. Yes, previous smoker; 3. Yes, current occasional smoker; 4. Yes, current regular smoker | **هل تدخن السجائر؟**  لا؛ نعم، مدخن سابق؛ نعم، مدخن حالي غير منتظم (في المناسبات)؛ نعم، مدخن حالي منتظم |
| --- | --- | --- |
|  | **Number of cigarettes smoked per day (Put 0 if not applicable):** | **عدد السجائر التي تدخنها يومياً (ضع 0 إذا لا ينطبق):** |
|  | **How old were you when you smoked your first cigarette?** | **كم كان عمرك عندما دخنت أول سيجارة؟** |
|  | **Do you consider yourself dependent to cigarettes?**   1. No, 2. probably not, 3. maybe, 4. probably yes, 5. Yes | **هل تعتبر نفسك مدمنا على السجائر؟**  لا، ربما لا، ربما، ربما نعم، نعم |
|  | **Are you a nargileh/waterpipe smoker?**   1. No; 2. Yes, previous smoker; 3. Yes, current occasional smoker; 4. Yes, current regular smoker | **هل تدخن النرجيلة؟**  لا؛ نعم، مدخن سابق؛ نعم، مدخن حالي غير منتظم (في المناسبات)؛ نعم، مدخن حالي منتظم |
|  | **Number of nargileh smoked per week? (Put 0 if not applicable):** | **عدد النرجيلة التي تدخنها في الأسبوع (ضع 0 إذا لا ينطبق):** |
|  | **How old were you when you smoked your first nargileh?** | **كم كان عمرك عندما دخنت أول نرجيلة؟** |
|  | **Do you consider yourself dependent to nargileh/waterpipe**?   1. No, 2. probably not, 3. maybe, 4. probably yes, 5. Yes | **هل تعتبر نفسك مدمنا على النرجيلة ؟**  لا، ربما لا، ربما، ربما نعم، نعم |
|  | **Number of smokers at home** | عدد المدخنين في المنزل |
|  | **Number of smokers at work (Put 0 if not applicable):** | عدد المدخنين في العمل **(ضع 0 إذا لا ينطبق):** |
|  | **Do you agree with banning smoking in cafés?**  1. Strongly disagree,  2. disagree,  3.neutral,  4.agree,  5.strongly agree | **هل توافق على منع التدخين في المقاهي؟**  أختلف بشدة، أختلف، محايد، أوافق، أوافق بشدة |
|  | **Do you agree with banning smoking for minors?**  Strongly disagree, disagree, neutral, agree, strongly agree | **هل توافق على منع التدخين للقُصَّر؟**  أختلف بشدة، أختلف، محايد، أوافق، أوافق بشدة |
|  | **Do you drink alcohol?**   1. No; 2. Yes, previous consumer; 3. Yes, current regular consumer; 4. Yes, occasional consumer | **هل تشرب الكحول؟**  **لا؛** نعم، مستهلك سابق؛ نعم، مستهلك حالي |
|  | **How many glasses of alcohol do you drink on one occasion? (Put 0 if not applicable):** | **كم كأس من الكحول تشرب في مناسبة واحدة؟ (ضع 0 إذا لا ينطبق):** |
|  | **Do you consider yourself dependent to alcohol?**   1. No; 2. Probably not; 3. Maybe; 4. Probably yes; 5. Yes | **هل تعتبر نفسك مدمناً على الكحول؟**  لا؛ ربما لا؛ ربما؛ ربما نعم؛ نعم |
|  | **Do you consume alcohol while smoking?**   1. Never; 2. Sometimes; 3. Most of the times; 4. Always 5. Not Applicable | **هل تستهلك الكحول أثناء التدخين؟**  أبداً؛ بعض الأحيان؛ معظم الأوقات؛ دائماً |
|  | **Do you consume caffeinated beverages (coffee and derivatives or energy drinks)?**   1. No; 2. Yes, previous consumer; 3. Yes, current consumer. | **هل تتناول المشروبات التي تحتوي على الكافيين (لقهوة ومشتقاتها أو مشروبات الطاقة)؟**  **لا؛** نعم، مستهلك سابق؛ نعم، مستهلك حالي |
|  | **How many cups of caffeinated beverages do you drink per day (Put 0 if not applicable):** | **كم كوب من المشروبات التي تحتوي على الكافيين تتناول يومياً (ضع 0 إذا لا ينطبق):** |
|  | **Do you consume caffeinated beverages while smoking?**  1. Never;  2. Sometimes;  3. Most of the times;  4.Always  5. Not applicable | **هل تستهلك المشروبات التي تحتوي على الكافيين أثناء التدخين؟**  أبداً؛ بعض الأحيان؛ معظم الأوقات؛ دائماً |

1. **The Lebanese Cigarette Dependence Score (LCD)**

**درجة إدمان اللبنانيين على السجائر**

| **1.** I smoke for conviviality  1. Yes, sure;  2. Yes, probably;  3. Yes, maybe;  0. No  4.NA | أدخن للمسايرة:  نعم، أكيد؛ نعم على الأرجح؛ نعم، ربما؛ كلا |
| --- | --- |
| **2.** I smoke to increase morale  Yes, sure; Yes, probably; Yes, maybe; No | أدخن لرفع المعنويات:  نعم، أكيد؛ نعم على الأرجح؛ نعم، ربما؛ كلا |
| **3.** I smoke for pleasure  Yes, sure; Yes, probably; Yes, maybe; No | أدخن للّذة:  نعم، أكيد؛ نعم على الأرجح؛ نعم، ربما؛ كلا |
| **4.** I smoke to concentrate while working  Yes, sure; Yes, probably; Yes, maybe; No | أدخن للتركيز عندما أعمل:  نعم، أكيد؛ نعم على الأرجح؛ نعم، ربما؛ كلا |
| **5.** I smoke to decrease nervousness  Yes, sure; Yes, probably; Yes, maybe; No | أدخن لتخفيف التوتر العصبي:  نعم، أكيد؛ نعم على الأرجح؛ نعم، ربما؛ كلا |
| **6.** Do you usually smoke alone?  1. Yes, always;  2. Yes, most of the times;  3. Yes, sometimes;  0. No  4. NA | هل تدخن عادةً بمفردك؟  نعم، دائماً؛ نعم، أكثر الأوقات؛ نعم، أحياناً؛ كلا |
| **7.** How soon after you wake up do you smoke your first cigarette?  1. 5 minutes;  2. 6–30 minutes;  3. 31–60minutes;  4. More than 60 minutes;  5. I only smoke occasionally  6.NA | بعد كم من الوقت من استيقاظك من النوم تدخن؟  5 دقائق؛ 6–30 دقيقة؛ 31–60 دقيقة؛ بعد أكثر من ستين دقيقة؛ لا أدخن إلا في المناسبات |
| **8.** Are you ready to leave your family on a holiday to go and search for cigarettes?  Yes, sure; Yes, probably; Yes, maybe; No | إذا كنت في يوم عطلة ولم يبق لديك سجائر، هل تترك عائلتك و تذهب للبحث عنها؟  نعم، أكيد؛ نعم على الأرجح؛ نعم، ربما؛ كلا |
| **9.** Do you prefer smoking cigarettes over going to the theatre, or movies or other activities?  Yes, sure; Yes, probably; Yes, maybe; No | هل تفضل التدخين على الذهاب إلى السينما أو المسرح أو ممارسة الرياضة؟  نعم، أكيد؛ نعم على الأرجح؛ نعم، ربما؛ كلا |
| **10.** Do you smoke if you are so ill and bedridden?  Yes, sure; Yes, probably; Yes, maybe; No | هل تدخن إذا كنت مريضا طريح الفراش؟  نعم، أكيد؛ نعم على الأرجح؛ نعم، ربما؛ كلا |
| **11.** Do you find it difficult to refrain from smoking wherever it is forbidden?  1.Yes, too much;  2.Yes, somehow;  3.Yes, a little bit;  0.No, not at all  4.NA | هل تنزعج من منع التدخين في بعض الحالات؟ (سينما، طائرة)  نعم كثيراً؛ نعم بعض الشيء؛ نعم قليلاً؛ كلا أبداً |
| **12.** Number of days you could stop smoking cigarettes  1.One day or less;  2.2–3 days;  3.4–7 days;  4.More than 7 days  5.Na | كم من الأيام يمكن أن تمضي من دون أن تدخن؟  يوم أو أقل؛ يومين أو ثلاثة؛ أربعة أيام إلى أسبوع؛ أكثر من أسبوع |
| **13.** How much time are you ready to spend searching for cigarettes?  1.<30 minutes;  2.30 minutes–2 hours;  3.Almost half a day;  4.A day or more  5.NA | كم من الوقت يمكن أن تمضي في البحث عن سجائر؟  أقل من نصف ساعة؛ تصف ساعة الى ساعتين؛ نصف نهار؛ يوم أو أكثر |
| **14.** How much money, in proportion to your income, are you ready to pay to buy cigarettes?  1.1% of your income or less;  2.2–10% of your income;  3.10–50% of your income;  4.More than 50% of your income  5.NA | كم من المال أنت مستعد لدفع ثمن نفس سيجارة عند الحاجة؟  1% من مدخولك أو أقل؛ 2–10% من مدخولك؛ 11–50% من مدخولك؛ أكثر من 50% من مدخولك |

1. **The Lebanese Waterpipe Dependence Scale (LWDS-11)**

**المقياس اللبناني للإدمان على الشيشة**

| **1.** Number of waterpipes you usually smoke per week  1. Less than 1;  2. 1–2;  3. 2–6;  4. More than 7  5. NA | كم نرجيلة تدخن حاليا في الأسبوع؟  أقل من واحدة؛ 1–2؛ 2–6؛ 7 أو أكثر |
| --- | --- |
| **2.** Do you smoke waterpipe alone?  1. No;  2. Yes, sometimes;  3. Yes, most of the times;  4. Yes, always | هل تدخن عادة بمفردك؟  كلا؛ نعم، أحياناً؛ نعم، أكثر الأوقات؛ نعم، دائماً |
| **3.** Do you smoke waterpipe when you are seriously ill?  1. No;  2. Yes, maybe;  3. Yes, probably;  4. Yes, absolutely | هل تدخن  النرجيلة إذا كنت مريضاً طريح الفراش؟  كلا؛ نعم، ربما؛ نعم، على الأرجح؛ نعم، أكيد |
| **4.** Number of days you could spend without waterpipe  1. More than 7 days;  2. 4 days–1 week;  3. 2–3 days;  4. 1 day or less | كم من الأيام يمكن أن تمضي من دون أن تدخن النرجيلة؟  أكثر من أسبوع؛ أربعة أيام إلى أسبوع؛ يومين أو ثلاثة؛ يوم أو أقل |
| **5.** Number of times you could stop waterpipe for more than 7 days?  1. Never;  2. Once;  3. Several times;  4. It always happens | كم من المرات استطعت أن توقف تدخين النرجيلة لأكثر من سبعة أيام؟  ولا مرة؛ مرة واحدة؛ عدة مرات؛ دائما يتكرر ذلك |
| **6.** What percentage of your monthly income would you spend for waterpipe smoking?  1. 1% of your income or less;  2. 2–10% of your income;  3. 10–50% of your income;  4. More than 50% of your income | كم من المال أنت مستعد لدفع ثمن نفس نرجيلة عند الحاجة؟  0–1% من مدخولك أو أقل؛ 2–10% من مدخولك؛ 11–50% من مدخولك؛ أكثر من 50% من مدخولك |
| **7.** Are you ready not to eat in exchange for a waterpipe?  1. No;  2. Yes, maybe;  3. Yes, probably;  4. Yes, absolutely | هل أنت مستعد لعدم الأكل مقابل الحصول على نفس نرجيلة؟  كلا؛ نعم، ربما؛ نعم، على الأرجح؛ نعم، أكيد |
| **8.** You smoke waterpipe to please others (for conviviality)  No; Yes, maybe; Yes, probably; Yes, absolutely | تدخن النرجيلة للمسايرة  كلا؛ نعم، ربما؛ نعم، على الأرجح؛ نعم، أكيد |
| **9.** You smoke waterpipe to improve your morale/mood  No; Yes, maybe; Yes, probably; Yes, absolutely | تدخن النرجيلة لرفع المعنويات  كلا؛ نعم، ربما؛ نعم، على الأرجح؛ نعم، أكيد |
| **10.** You smoke waterpipe for pleasure  No; Yes, maybe; Yes, probably; Yes, absolutely | تدخن النرجيلة للّذة  كلا؛ نعم، ربما؛ نعم، على الأرجح؛ نعم، أكيد |
| **11.** You smoke waterpipe to relax your nerves  No; Yes, maybe; Yes, probably; Yes, absolutely | تدخن النرجيلة لتخفيف التوتر العصبي  كلا؛ نعم، ربما؛ نعم، على الأرجح؛ نعم، أكيد |

1. **The Alcohol Use Disorders Identification Test (AUDIT)**

**اختبار التعرف على اضطرابات تعاطي الكحول**

| **1.** How often do you have a drink containing alcohol?  Never (0 points)  Monthly or less (1 point)  2 to 4 times per month (2 points)  2 to 3 times per week (3 points)  4 or more times per week (4 points)  5 NA | كم مرة تشرب؟  أبداً  مرة واحدة في الشهر أو أقل  2 إلى 4 مرات في الشهر  2 إلى 3 مرات في الأسبوع  4 مرات على الأقل في الأسبوع |
| --- | --- |
| **2.** How many units of alcohol do you drink on a typical day when you are drinking?  0 to 2 (0 points)  3 to 4 (1 point)  5 to 6 (2 points)  7 to 9 (3 points)  10 or more (4 points)   1. **NA** | كم عدد المشروبات التي تحتوي على الكحول تستهلك في يوم نموذجي عندما تشرب؟  من 0 إلى 2  من 3 إلى 4  من 5 إلى 6  من 7 إلى 9  10 أو أكثر |
| **3.** How often have you had 6 or more drinks on one occasion?  Never (0 points)  Less than monthly (1 point)  Monthly (2 points)  Weekly (3 points)  Daily or almost daily (4 points)  **5.NA** | كم مرة تشرب ستة كؤوس أو أكثر من المشروبات في مناسبة معينة؟  أبداً  أقل من مرة في الشهر  مرة واحدة في الشهر  مرة واحدة في الأسبوع  كل يوم أو ما يقاربه |
| **4.** How often during the last year have you found that you were not able to stop drinking once you had started?  Never (0 points)  Less than monthly (1 point)  Monthly (2 points)  Weekly (3 points)  Daily or almost daily (4 points)  5.NA | في العام الماضي، كم مرة وجدت نفسك غير قادر عن التوقف عن الشرب بمجرد أن تبدأ بتناول الكؤوس؟  أبداً  أقل من مرة في الشهر  مرة واحدة في الشهر  مرة واحدة في الأسبوع  كل يوم أو ما يقاربه |
| **5.** How often during the last year have you failed to do what was normally expected from you because of your drinking?  Never (0 points)  Less than monthly (1 point)  Monthly (2 points)  Weekly (3 points)  Daily or almost daily (4 points) | في العام الماضي، كم مرة كان الشرب الخاص بك يمنعك من القيام بما كان متوقّعاً منك القيام به؟  أبداً  أقل من مرة في الشهر  مرة واحدة في الشهر  مرة واحدة في الأسبوع  كل يوم أو ما يقاربه |
| **6.** How often during the last year have you needed an alcoholic drink in the morning to get yourself going after a heavy drinking session?  Never (0 points)  Less than monthly (1 point)  Monthly (2 points)  Weekly (3 points)  Daily or almost daily (4 points) | في العام الماضي، كم عدد المرات التي كنت في حاجة الى تناول الشراب الأول، بعد شرب الكثير في اليوم السابق كي تتمكّن من المباشرة بنشاطك اليومي؟  أبداً  أقل من مرة في الشهر  مرة واحدة في الشهر  مرة واحدة في الأسبوع  كل يوم أو ما يقاربه |
| **7.** How often during the last year have you had a feeling of guilt or remorse after drinking?  Never (0 points)  Less than monthly (1 point)  Monthly (2 points)  Weekly (3 points)  Daily or almost daily (4 points) | في العام الماضي، كم مرة كان لديك مشاعر الذنب أو الندم بعد الشرب؟  أبداً  أقل من مرة في الشهر  مرة واحدة في الشهر  مرة واحدة في الأسبوع  كل يوم أو ما يقاربه |
| **8.** How often during the last year have you been unable to remember what happened the night before because you had been drinking?  Never (0 points)  Less than monthly (1 point)  Monthly (2 points)  Weekly (3 points)  Daily or almost daily (4 points) | في العام الماضي، كم مرة كنت غير قادر على تذكر ما حدث في الليلة السابقة لأنك قد أفرطت في تناول المشروب؟  أبداً  أقل من مرة في الشهر  مرة واحدة في الشهر  مرة واحدة في الأسبوع  كل يوم أو ما يقاربه |
| **9.** Have you or somebody else been injured as a result of your drinking?  No (0 points)  Yes, but not in the last year (2 points)  Yes, during the last year (4 points)  5.NA | هل أصبت أو أصيب أحدهم لأنك كنت تشرب؟  لا  نعم، ولكن ليس في العام الماضي  نعم، في السنة الحالية |
| **10.** Has a relative or friend or a doctor or other health worker been concerned about your drinking or suggested that you cut down?  No (0 points)  Yes, but not in the last year (2 points)  Yes, during the last year (4 points) | ھل کان أحد الوالدین أو الأصدقاء أو الطبیب أو غیرھم من مقدمي الرعایة قلقین بشأن الشرب أو اقترح أن تقللھا؟  لا  نعم، ولكن ليس في العام الماضي  نعم، في السنة الحالية |

# SOCIAL DESIRABILITY

# الاستحسان الاجتماعي

1. **The Marlowe-Crowne Social Desirability Scale**

**مقياس مارلو - كراون للاستحسان الاجتماعي**

Please read the below statements and indicate your agreement with each on the following scale: strongly disagree (1); disagree (2); neutral (3); agree (4); strongly agree (5)

يرجى قراءة العبارات أدناه والإشارة إلى موافقتك على كل منها على المقياس التالي: غير موافق بشدة (1)؛ غير موافق (2)؛ محايد (3)؛ موافق (4)؛ موافق بشدة (5)

| **1.** Before voting, I thoroughly investigate the qualifications of all the candidates | قبل التصويت، أقوم بالتحقق بدقة من مؤهلات جميع المرشحين |
| --- | --- |
| **2.** I never hesitate to go out of my way to help someone in trouble | لا أتردد أبدًا في بذل قصارى جهدي لمساعدة شخص في ورطة |
| **3.** It is sometimes hard for me to go on with my work if I am not encouraged | أحيانًا يكون من الصعب عليّ الاستمرار في عملي إذا لم يتم تشجيعي |
| **4.** I have never intensely disliked anyone | لم أكره أي شخص بشدة |
| **5.** On occasion, I have had doubts about my ability to succeed in life | في بعض الأحيان، كانت لدي شكوك حول قدرتي على النجاح في الحياة |
| **6.** I sometimes feel resentful when I don’t get my way | أشعر أحيانًا بالاستياء عندما لا أحصل على ما أريد |
| **7.** I am always careful about my manner of dress | أنا دائمًا حريص بشأن طريقة لباسي |
| **8.** My table manners at home are as good as when I eat out in a restaurant | آداب المائدة في المنزل جيدة كما هي عندما أتناول الطعام في الخارج في مطعم |
| **9.** If I could get into a movie without paying and be sure I was not seen I would probably do it | إذا كان بإمكاني الذهاب إلى فيلم دون دفع والتأكد من عدم رؤيتي، فربما أفعل ذلك |
| **10.** On a few occasions, I have given up doing something because I thought too little of my ability | في بعض المناسبات، تخليت عن القيام بشيء ما لأنني اعتقدت أن قدراتي قليلة جدًا |
| **11.** I like to gossip at times | أحب النميمة في بعض الأحيان |
| **12.** There have been times when I felt rebelling against people in authority even though I know they were right | كانت هناك أوقات شعرت فيها بالتمرد ضد الأشخاص ذوي السلطة على الرغم من أنني أعلم أنهم على حق |
| **13.** No matter who I am talking to, I am always a good listener | بغض النظر عمن أتحدث إليه، فأنا دائمًا مستمع جيد |
| **14.** I can remember “playing sick” to get out of something | أتذكر أنني كنت "أتظاهر بالمرض" لأتخلص من شيء ما |
| **15.** There have been occasions when I took advantage of someone | كانت هناك مناسبات استغليت فيها شخصًا ما |
| **16.** I am always willing to admit it when I make a mistake | أنا دائمًا على استعداد للاعتراف بذلك عندما أرتكب خطأ |
| **17.** I always try to practice when I preach | أحاول دائمًا التدرب عندما أكرز |
| **18.** I don’t find it particularly difficult to get along with loud-mouthed, obnoxious people (T) | لا أجد صعوبة كبيرة في التعامل مع الأشخاص الفظين والمزعجين |
| **19.** I sometimes try to get even rather than forgive and forget | أحاول أحيانًا الانتقام بدلاً من المسامحة والنسيان |
| **20.** When I don’t know something, I don’t at all mind admitting it | عندما لا أعرف شيئًا، لا أمانع على الإطلاق في الاعتراف به |
| **21.** I am always courteous, even to people who are disagreeable | أنا دائمًا مهذب، حتى مع الأشخاص غير المرغوب فيهم |
| **22.** At times I have really insisted on having things my own way | أصررت في بعض الأحيان على أن تكون الأمور على طريقتي |
| **23.** There have been occasions when I felt like smashing things (F) | كانت هناك مناسبات شعرت فيها برغبة في تحطيم الأشياء |
| **24.** I would never think of letting someone else be punished for my wrongdoings | لم أفكر أبدًا في السماح لشخص آخر بمعاقبته على أخطائي |
| **25.** I never resent being asked to return a favor | لم أستاء أبدًا من مطالبة أحدهم برد الجميل |
| **26.** I have never been irked when people express ideas very different from my own | لم أشعر أبدًا بالانزعاج عندما يعبر الناس عن أفكار مختلفة تمامًا عن أفكاري |
| **27.** I never make a long trip without checking the safety of my car | لا أقوم أبدًا برحلة طويلة دون التحقق من سلامة السيارة |
| **28.** There have been times when I was quite jealous of the good fortune of others | كانت هناك أوقات كنت أشعر فيها بالغيرة الشديدة من حسن حظ الآخرين |
| **29.** I have almost never felt the urge to tell someone off | لم أشعر أبدًا بالحاجة إلى توبيخ شخص ما |
| **30.** I am sometimes irritated by people who ask favors of me | أشعر أحيانًا بالانزعاج من الأشخاص الذين يطلبون مني معروفًا |
| **31.** I have never felt that I was punished without cause | لم أشعر أبدًا بأنني عوقبت دون سبب |
| **32.** I sometimes think when people have a misfortune they only got what they deserved | أفكر أحيانًا عندما يتعرض الناس لسوء حظ فإنهم ينالون ما يستحقونه فقط |
| **33.** I have never deliberately said something that hurt someone’s feelings | لم أقل عمدًا شيئًا يؤذي مشاعر شخص ما |

1. **The Balanced Inventory of Desirable Responding**

**الجرد المتوازن للاستجابة المستحسنة**

Please rate the below statements from 1 (not true) to 7 (very true)

يرجى تقييم العبارات التالية من 1 (غير صحيح) إلى 7 (صحيح جداً)

| **1.** My first impressions of people usually turn out to be right. | انطباعاتي الأولى عن الناس عادة ما تكون صحيحة. |
| --- | --- |
| **2.** It would be hard for me to break any of my bad habits. | من الصعب عليّ أن أتخلص من أي من عاداتي السيئة. |
| **3.** I don't care to know what other people really think of me. | لا أهتم بمعرفة رأي الآخرين فيّ. |
| **4.** I have not always been honest with myself. | لم أكن صادقًا مع نفسي دائمًا. |
| **5.** I always know why I like things. | أعرف دائمًا سبب إعجابي بالأشياء. |
| **6.** When my emotions are aroused, it biases my thinking. | عندما تثار مشاعري، فإن ذلك يؤثر على تفكيري. |
| **7.** Once I've made up my mind, other people can seldom change my opinion. | بمجرد أن أتخذ قراري، نادرًا ما يغير الآخرون رأيي. |
| **8.** I am not a safe driver when I exceed the speed limit. | لست سائقًا آمنًا عندما أتجاوز حد السرعة. |
| **9.** I am fully in control of my own fate. | أنا أتحكم تمامًا في مصيري. |
| **10.** It's hard for me to shut off a disturbing thought. | من الصعب عليّ أن أتوقف عن التفكير المزعج. |
| **11.** I never regret my decisions. | لا أندم أبدًا على قراراتي. |
| **12.** I sometimes lose out on things because I can't make up my mind soon enough. | أحيانًا أخسر أشياء لأنني لا أستطيع اتخاذ قراري في الوقت المناسب. |
| **13.** The reason I vote is because my vote can make a difference. | السبب الذي يجعلني أصوت هو أن صوتي يمكن أن يحدث فرقًا. |
| **14.** My parents were not always fair when they punished me. | لم يكن والداي منصفين دائمًا عندما عاقباني. |
| **15.** I am a completely rational person. | أنا شخص عقلاني تمامًا. |
| **16.** I rarely appreciate criticism. | نادرًا ما أقدر النقد. |
| **17.** I am very confident of my judgments | أنا واثق جدًا من أحكامي |
| **18.** I have sometimes doubted my ability as a lover. | أشك أحيانًا في قدرتي على الحب. |
| **19.** It's all right with me if some people happen to dislike me. | لا بأس معي إذا كرهني بعض الأشخاص. |
| **20.** I don't always know the reasons why I do the things I do. | لا أعرف دائمًا الأسباب التي تجعلني أفعل الأشياء التي أفعلها. |
| **21.** I sometimes tell lies if I have to. | أكذب أحيانًا إذا اضطررت إلى ذلك. |
| **22.** I never cover up my mistakes. | لا أتستر على أخطائي أبدًا. |
| **23.** There have been occasions when I have taken advantage of someone. | كانت هناك مناسبات استغليت فيها شخصًا ما. |
| **24.** I never swear. | لا أقول أبدًا كلاما بذيئا. |
| **25.** I sometimes try to get even rather than forgive and forget. | أحاول أحيانًا أن أنتقم بدلاً من أن أسامح وأنسى. |
| **26.** I always obey laws, even if I'm unlikely to get caught. | أطيع القوانين دائمًا، حتى لو كان من غير المرجح أن يتم القبض علي. |
| **27.** I have said something bad about a friend behind his/her back. | قلت شيئًا سيئًا عن صديق خلف ظهره. |
| **28.** When I hear people talking privately, I avoid listening. | عندما أسمع الناس يتحدثون على انفراد، أتجنب الاستماع. |
| **29.** I have received too much change from a salesperson without telling him or her. | تلقيت الكثير من النقود من بائع دون أن أخبره. |
| **30.** I always declare everything at customs. | أعلن دائمًا عن كل شيء في الجمارك. |
| **31.** When I was young I sometimes stole things. | عندما كنت صغيرًا، كنت أسرق أشياءً أحيانًا. |
| **32.** I have never dropped litter on the street. | لم أسقط القمامة في الشارع أبدًا. |
| **33.** I sometimes drive faster than the speed limit. | كنت أقود السيارة أحيانًا بسرعة أكبر من الحد الأقصى للسرعة. |
| **34.** I never read sexy books or magazines. | لم أقرأ الكتب أو المجلات الجنسية أبدًا. |
| **35.** I have done things that I don't tell other people about. | فعلت أشياء لم أخبر بها الآخرين. |
| **36.** I never take things that don't belong to me. | لم آخذ أبدًا أشياء لا تخصني. |
| **37.** I have taken sick-leave from work or school even though I wasn't really sick. | أخذت إجازة مرضية من العمل أو المدرسة على الرغم من أنني لم أكن مريضًا حقًا. |
| **38.** I have never damaged a library book or store merchandise without reporting it. | لم أتلف أبدًا كتابًا من مكتبة أو بضائع متجر دون الإبلاغ عن ذلك. |
| **39.** I have some pretty awful habits. | لدي بعض العادات السيئة جدًا. |
| **40.** I don't gossip about other people's business. | لا أتحدث عن أعمال الآخرين. |

1. **The Social Desirability Scale-17**

**مقياس الاستحسان الاجتماعي**

Please read the below statements and indicate your agreement with each on the following scale: strongly disagree (1); disagree (2); neutral (3); agree (4); strongly agree (5)

يرجى قراءة العبارات أدناه والإشارة إلى موافقتك على كل منها على المقياس التالي: غير موافق بشدة (1)؛ غير موافق (2)؛ محايد (3)؛ موافق (4)؛ موافق بشدة (5)

| **1.** I sometimes litter. | أقوم أحيانًا برمي النفايات. |
| --- | --- |
| **2.** I always admit my mistakes openly and face the potential negative consequences. | أعترف دائمًا بأخطائي علنًا وأواجه العواقب السلبية المحتملة. |
| **3.** In traffic I am always polite and considerate of others. | في حركة المرور، أكون مهذبًا ومراعيًا للآخرين دائمًا. |
| **4.** I have tried illegal drugs (for example, marijuana, cocaine, etc.). | جربت المخدرات غير المشروعة (على سبيل المثال، الماريجوانا والكوكايين وما إلى ذلك). |
| **5.** I always accept others' opinions, even when they don't agree with my own. | أتقبل دائمًا آراء الآخرين، حتى عندما لا تتفق مع آرائي. |
| **6.** I take out my bad moods on others now and then. | أفرغ مزاجي السيئ على الآخرين بين الحين والآخر. |
| **7.** There has been an occasion when I took advantage of someone else. | كانت هناك مناسبة استغليت فيها شخصًا آخر. |
| **8.** In conversations, I always listen attentively and let others finish their sentences. | في المحادثات، أستمع دائمًا باهتمام وأدع الآخرين يكملون جملتهم. |
| **9.** I never hesitate to help someone in case of emergency. | لا أتردد أبدًا في مساعدة شخص ما في حالة الطوارئ. |
| **10.** When I have made a promise, I keep it--no ifs, ands or buts. | عندما أقطع وعدًا، أفي به - بدون شروط أو استثناءات. |
| **11.** I occasionally speak badly of others behind their back. | أتحدث أحيانًا بشكل سيء عن الآخرين خلف ظهورهم. |
| **12.** I would never live off other people. | لن أعيش على حساب الآخرين أبدًا. |
| **13.** I always stay friendly and courteous with other people, even when I am stressed out. | أظل دائمًا ودودًا ومهذبًا مع الآخرين، حتى عندما أكون متوترًا. |
| **14.** During arguments, I always stay objective and matter-of-fact. | أثناء الجدال، أظل دائمًا موضوعيًا وواقعيًا. |
| **15.** There has been at least one occasion when I failed to return an item that I borrowed. | كانت هناك مناسبة واحدة على الأقل فشلت فيها في إرجاع شيء استعرته. |
| **16.** I always eat a healthy diet. | أتناول دائمًا نظامًا غذائيًا صحيًا. |
| **17.** Sometimes, I only help because I expect something in return. | في بعض الأحيان أساعد فقط لأنني أتوقع شيئًا في المقابل. |

1. **The Conformity Scale**

**مقياس المطابقة**

Please rate the below statements from 1 (not at all true) to 7 (extremely true)

يرجى تقييم العبارات التالية من 1 (غير صحيح على الإطلاق) إلى 7 (صحيح للغاية)

| **1.** I often rely on and act upon the advice of others. | أعتمد على نصائح الآخرين وأتصرف بناءً عليها في كثير من الأحيان. |
| --- | --- |
| **2.** I would be the last one to change my opinion in a heated argument on a controversial topic.* | أكون آخر من يغير رأيه في نقاش حاد حول موضوع مثير للجدل.* |
| **3.** Generally, I’d rather give in and go along for the sake of peace than struggle to have it my way. | بشكل عام، أفضل الاستسلام والمضي قدمًا من أجل السلام بدلاً من النضال من أجل تحقيق ما أريد. |
| **4.** I tend to follow family traditions in making political decisions. | أميل إلى اتباع التقاليد العائلية في اتخاذ القرارات السياسية. |
| **5.** Basically, my friends are the ones who decide what we do together. | في الأساس، أصدقائي هم من يقررون ما نفعله معًا. |
| **6.** A charismatic and eloquent speaker can easily influence and change my ideas. | يمكن للمتحدث الكاريزماتي والبليغ التأثير على أفكاري وتغييرها بسهولة. |
| **7.** I am more independent than conforming in my ways.* | أنا أكثر استقلالية من التقيد بطريقتي.* |
| **8.** If someone is very persuasive, I tend to change my opinion and go along with them. | إذا كان شخص ما مقنعًا للغاية، فأنا أميل إلى تغيير رأيي والمضي قدمًا معه. |
| **9.** I don’t give in to others easily.* | لا أستسلم للآخرين بسهولة.* |
| **10.** I tend to rely on others when I have to make an important decision quickly. | أميل إلى الاعتماد على الآخرين عندما يتعين علي اتخاذ قرار مهم بسرعة. |
| **11.** I prefer to find my own way in life rather than find a group I can follow.* | أفضل أن أجد طريقي الخاص في الحياة بدلاً من العثور على مجموعة يمكنني اتباعها* |

**Additional questions**

Please read the below statements and indicate your agreement with each on the following scale: strongly disagree (1); disagree (2); neutral (3); agree (4); strongly agree (5)

يرجى قراءة العبارات أدناه والإشارة إلى موافقتك على كل منها على المقياس التالي: غير موافق بشدة (1)؛ غير موافق (2)؛ محايد (3)؛ موافق (4)؛ موافق بشدة (5)

|  | In terms of opinion, I prefer to blend in with the majority rather than have my own opinion | من حيث الرأي، أفضل أن أختلط بالأغلبية بدلاً من أن يكون لي رأيي الخاص |
| --- | --- | --- |
|  | In a discussion, I prefer to blend in with the majority rather than voice my own opinion | في المناقشة، أفضل أن أختلط بالأغلبية بدلاً من التعبير عن رأيي الخاص |
|  | If I am asked about my opinion on a sensitive topic, I would lie if I knew that my opinion does not fit with the cultural norm | إذا سئلت عن رأيي في موضوع حساس، فسأكذب إذا علمت أن رأيي لا يتناسب مع المعايير الثقافية |
|  | In terms of behavior, it is important for me to act in a way that does not deceive my family | من حيث السلوك، من المهم بالنسبة لي أن أتصرف بطريقة لا تخدع عائلتي |
|  | In terms of behavior, it is important for me to act in a way that does not deceive my friends | من حيث السلوك، من المهم بالنسبة لي أن أتصرف بطريقة لا تخدع أصدقائي |
|  | In terms of behavior, it is important for me to act according to the social norms | من حيث السلوك، من المهم بالنسبة لي أن أتصرف وفقًا للمعايير الاجتماعية |
|  | In terms of behavior, it is important for me to act according to the religious norms | من حيث السلوك، من المهم بالنسبة لي أن أتصرف وفقًا للمعايير الدينية |
|  | I am a person who likes roots and traditions | أنا شخص يحب الجذور والتقاليد |
|  | I am a person who likes comfort zones | أنا شخص يحب مناطق الراحة |
|  | I am a person who likes strong sensations in life (high arousal sensations) | أنا شخص يحب الأحاسيس القوية في الحياة (أحاسيس الإثارة العالية) |
|  | One of my main goals in life has been to make my parents proud | أحد أهدافي الرئيسية في الحياة هو جعل والدي فخورين |
|  | I make a lot of effort to live up to what friends expect | أبذل الكثير من الجهد للعيش وفقًا لما يتوقعه الأصدقاء |
|  | Children should be encouraged to learn obedience at home | يجب تشجيع الأطفال على تعلم الطاعة في المنزل |
|  | I seek to be myself rather than to follow others | أسعى أن أكون نفسي بدلاً من أن أتبع الآخرين |
|  | I am a person who likes cool sensations (low arousal, relaxing, being in peace with my environment) | أنا شخص يحب الأحاسيس الهادئة (الإثارة المنخفضة، الاسترخاء، الشعور بالسلام مع بيئتي) |
