# Supplementary material for: Cluster analysis of conformity and social desirability: Association with use and problematic use of licit substances
Source: Addict Behav Rep. 2026 May 20;23:100710. doi: 10.1016/j.abrep.2026.100710 (PMC13223954; doi:10.1016/j.abrep.2026.100710)
Supplement: Supplementary file 2 — Supplementary material 2: Construct Validity and Reliability of the Lebanese Social Desirability Scale (LSDS) [file mmc2.docx]

**Supplementary file 3**

**The Lebanese Social Desirability Scale (LSDS) construct validity and reliability**

The 36 items that were included in the Exploratory Factor Analysis (EFA) and retained in the final structure loaded on three factors after varimax rotation (**Supplementary Table 1**): Factor 1 included 15 items, and represented perfectionist, virtuous, and impression management (Virtuous Perfectionism subscale). Factor 2 included 8 items, and was related to lower conscientiousness, lower impression management, and possibly higher openness to experience (Independent Thinking subscale). As for Factor 3 (13 items), it was about overall realistic, socially adaptive, lower honesty, and moderately self-serving tendencies (Socially Strategic Pragmatism subscale). The structure of the Lebanese Social Desirability Scale also showed excellent sampling adequacy, and the reliability of the subscales was very good to excellent. A Confirmatory Factor Analysis (CFA) was conducted to confirm unidimensionality, and its results were acceptable for 2 out of 4 criteria; thus, the three Factors were used separately as subscales.

| **Supplementary Table 1: Factor analysis of the Lebanese Social Desirability Scale (LSDS)** | | | | | | | | | |
| --- | --- | --- | --- | --- | --- | --- | --- | --- | --- |
| **Varimax rotated matrix** | | | | | | | | | |
| **Items** | | | | | **Factor 1** | **Factor 2** | | **Factor 3** | |
| I don't gossip about other people's business | | | | | 0.830 |  | |  | |
| I have never damaged a library book or store merchandise without reporting it | | | | | 0.818 |  | |  | |
| I never hesitate to help someone in case of an emergency | | | | | 0.807 |  | |  | |
| My parents were not always fair when they punished me | | | | | 0.787 |  | |  | |
| In traffic I am always polite and considerate of others | | | | | 0.779 |  | |  | |
| I never take things that don't belong to me | | | | | 0.777 |  | |  | |
| I always obey laws, even if I'm unlikely to get caught | | | | | 0.774 |  | |  | |
| I seek to be myself rather than to follow others | | | | | 0.768 |  | |  | |
| I always accept others' opinions, even when they don't agree with my own | | | | | 0.751 |  | |  | |
| I rarely appreciate criticism | | | | | 0.727 |  | |  | |
| My first impressions of people usually turn out to be right | | | | | 0.709 |  | |  | |
| It's all right with me if some people happen to dislike me | | | | | 0.693 |  | |  | |
| I always declare everything at customs | | | | | 0.670 |  | |  | |
| I have never been irked when people express ideas very different from my own | | | | | 0.656 |  | |  | |
| I never make a long trip without checking the safety of my car | | | | | 0.635 |  | |  | |
| I sometimes try to get even rather than forgive and forget | | | | |  | 0.759 | |  | |
| When I was young, I sometimes stole things | | | | |  | 0.759 | |  | |
| I have received too much change from a salesperson without telling him or her | | | | |  | 0.749 | |  | |
| I never read sexy books or magazines | | | | |  | 0.705 | |  | |
| I have taken sick leave from work or school even though I wasn't really sick | | | | |  | 0.658 | |  | |
| I have some pretty awful habits | | | | |  | 0.646 | |  | |
| I never regret my decisions | | | | |  | 0.624 | |  | |
| It would be hard for me to break any of my bad habits | | | | |  | 0.541 | |  | |
| I occasionally speak badly of others behind their back | | | | |  |  | | 0.719 | |
| There has been an occasion when I took advantage of someone else | | | | |  |  | | 0.665 | |
| Sometimes, I only help because I expect something in return | | | | |  |  | | 0.652 | |
| I have tried illegal drugs (for example, marijuana, cocaine, etc.). | | | | |  |  | | 0.623 | |
| There have been occasions when I took advantage of someone | | | | |  |  | | 0.600 | |
| I sometimes think that when people have a misfortune, they only get what they deserve | | | | |  |  | | 0.593 | |
| If I am asked about my opinion on a sensitive topic, I would lie if I knew that my opinion does not fit with the cultural norm | | | | |  |  | | 0.547 | |
| There have been times when I felt rebelling against people in authority, even though I know they were right | | | | |  |  | | 0.531 | |
| I make a lot of effort to live up to what friends expect | | | | |  |  | | 0.509 | |
| It is sometimes hard for me to go on with my work if I am not encouraged | | | | |  |  | | 0.488 | |
| I sometimes litter | | | | |  |  | | 0.486 | |
| In terms of opinion, I prefer to blend in with the majority rather than have my own opinion | | | | |  |  | | 0.486 | |
| I don’t find it particularly difficult to get along with loud-mouthed, obnoxious people | | | | |  |  | | 0.475 | |
| **Percentage variance explained = 55.75%** | | | | |  |  | |  | |
| **Cronbach alpha** | | | | | 0.945 | 0.747 | | 0.856 | |
| **Kaiser-Meyer-Olkin (KMO)= 0.931** | | | | |  |  | |  | |
| **Bartlett’s test of sphericity p<0.001** | | | | |  |  | |  | |
| Factor 1 (15 items): Virtuous Perfectionism; Factor 2 (8 items): Independent Thinking; Factor 3 (13 items): Socially Strategic Pragmatism. | | | | | | | | | |
| **Confirmatory factor analysis of the Lebanese Social Desirability Scale structure** | | | | | | | | | |
|  | **χ^2^** | **df** | **χ^2^/df** | **RMSEA (95% CI)** | | | **CFI** | | **TLI** |
| Lebanese Social Desirability Scale (LSDS) structure | 2835.103 | 591 | 4.79 | 0.104 (0.100, 0.108) | | | 0.734 | | 0.716 |
| Abbreviations: CFI: Comparative Fit Index; CI: Confidence Interval; RMSEA: Root Mean Square Error of Approximation; TLI: Tucker–Lewis Index. Acceptable cut-off values: Chi-square (χ^2^/df) < 2–5; RMSEA acceptable fit is considered for values <0.11; CFI and TLI acceptable values are ≥0.90. | | | | | | | | | |
