# Supplementary material for: Cluster analysis of conformity and social desirability: Association with use and problematic use of licit substances
Source: Addict Behav Rep. 2026 May 20;23:100710. doi: 10.1016/j.abrep.2026.100710 (PMC13223954; doi:10.1016/j.abrep.2026.100710)

**Clusters properties**

In particular, cluster-specific covariance structures indicated unequal variance across dimensions, consistent with anisotropic dispersion. This was further supported by exploratory low-dimensional projections (e.g., t-SNE), which showed elongated and partially overlapping cluster shapes rather than compact spherical groupings: visual inspection of the t-SNE projection suggested that clusters were not strictly spherical, as several groups displayed elongated and partially overlapping structures, indicating departures from isotropic dispersion in the feature space. This is theoretically expected as the items are correlated. It is thus more appropriate to interpret clusters as structured profiles rather than geometrically homogeneous groups, given evidence of anisotropic dispersion. We also run a Gaussian mixture model for sensitivity: Comparison between the original clustering solution and the Gaussian mixture model allowing for full covariance structures, showed moderate agreement (Adjusted Rand Index = 0.58). This suggests that while the initial clusters capture meaningful structure, they do not fully reflect the underlying anisotropic distribution of the data. The mixture model identified similar but more flexibly shaped groupings, consistent with correlated dimensions and unequal variance across factors.

**Elbow plot**

The mean distance of each case from its assigned cluster center was examined to evaluate cluster cohesion (Figure S1). Cluster 3 exhibited the lowest mean distance, indicating that its members were closely grouped around the cluster centroid and the cluster was relatively compact. In contrast, Cluster 1 had the highest mean distance, suggesting greater dispersion among its members. Clusters 2, 4, and 5 showed intermediate distances, reflecting moderate cohesion


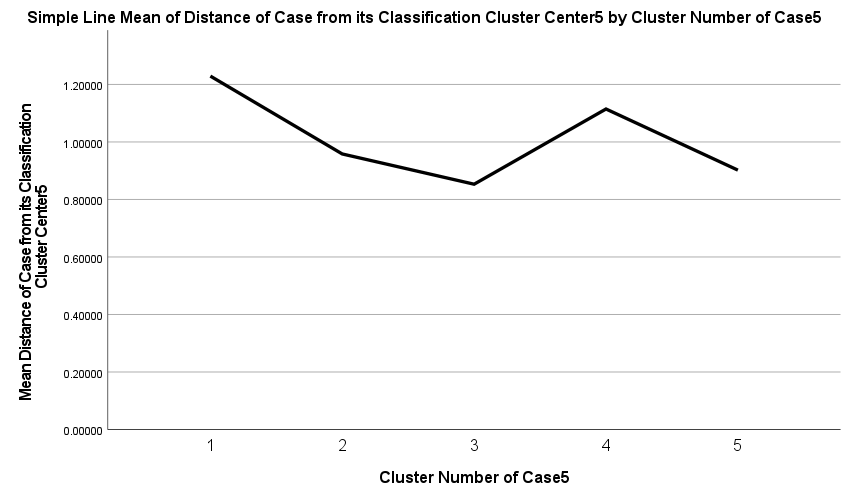


**Figure S1: Mean distance of each case from its assigned cluster center**

**Silhouette analysis**

Cluster quality was evaluated using Silhouette scores. The mean Silhouette score for the three clusters was 0.40 (SD = 0.20), with individual cluster means ranging from 0.29 to 0.49. Cluster 1 (n = 127) had a mean Silhouette of 0.29 (SD = 0.14), Cluster 2 (n = 143) had a mean of 0.35 (SD = 0.20), and Cluster 3 (n = 165) had the highest mean score of 0.49 (SD = 0.18), indicating the most cohesive cluster. The overall range of Silhouette scores was 0.01–0.89, showing that while most participants were moderately well-clustered, a few had low scores and were less clearly assigned to a cluster. Overall, these results suggest that the three-cluster solution provides a reasonable separation among participant profile

| **Table S1: Silhouette Scores for K-Means Cluster Solution** | | | | |
| --- | --- | --- | --- | --- |
| **Cluster** | **n** | **Mean Silhouette ± SD** | **Min** | **Max** |
| 1 | 127 | 0.29 ± 0.14 | 0.01 | 0.67 |
| 2 | 143 | 0.35 ± 0.20 | 0.01 | 0.89 |
| 3 | 165 | 0.49 ± 0.18 | 0.02 | 0.85 |
| **Overall** | 435 | 0.40 ± 0.20 | 0.01 | 0.89 |

**Normality assessment**

The three outcome variables were clearly non-normally distributed

| **Statistics** | | | | |
| --- | --- | --- | --- | --- |
|  | | LCD_scale | LWDS11_scale | AUDIT_scale |
| N | Valid | 197 | 169 | 108 |
|  | Missing | 263 | 291 | 352 |
| Mean | | 11.6599 | 8.5030 | 6.6667 |
| Std. Deviation | | 9.52500 | 6.29921 | 7.75682 |
| Skewness | | .715 | .786 | 1.519 |
| Std. Error of Skewness | | .173 | .187 | .233 |
| Kurtosis | | -.489 | -.342 | 1.285 |
| Std. Error of Kurtosis | | .345 | .371 | .461 |
| Minimum | | 1.00 | 1.00 | 1.00 |
| Maximum | | 36.00 | 26.00 | 34.00 |


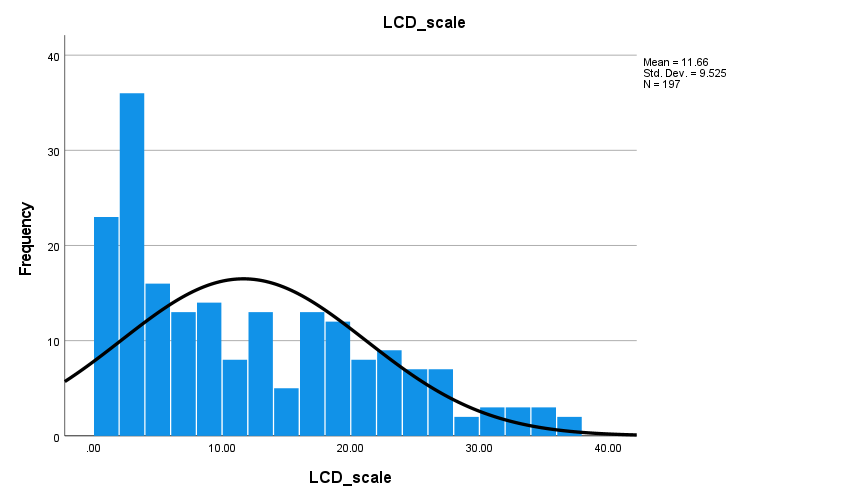


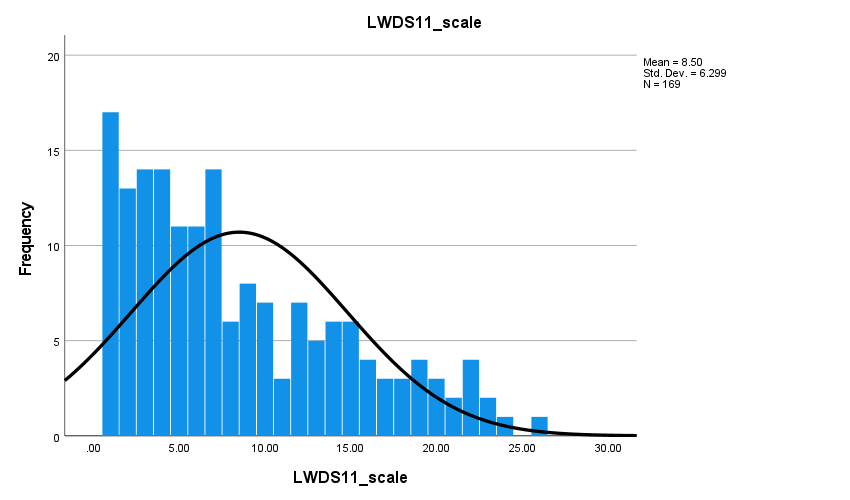


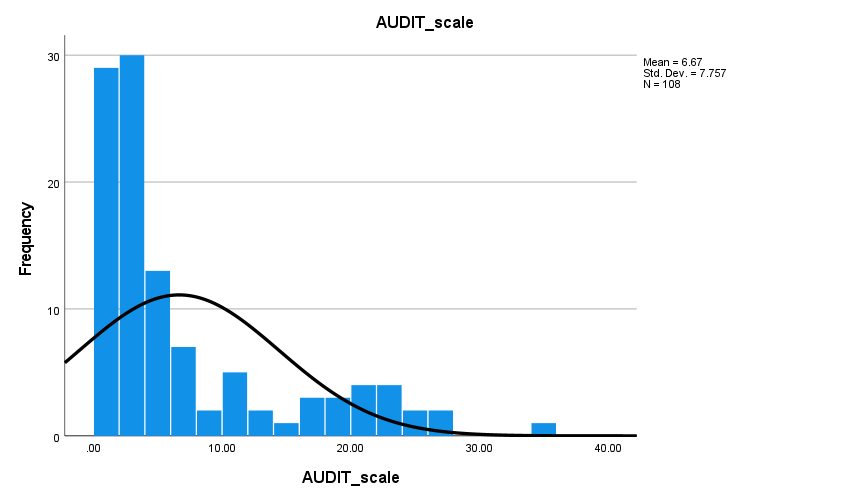

Supplement: Supplementary file 3 — Supplementary material 3: Cluster Properties and Normality Checking [file mmc3.docx]
